# Deciphering quinazoline derivatives’ interactions with EGFR: a computational quest for advanced cancer therapy through 3D-QSAR, virtual screening, and MD simulations

**DOI:** 10.3389/fphar.2024.1399372

**Published:** 2024-10-24

**Authors:** Sirajudheen Anwar, Jowaher Alanazi, Nafees Ahemad, Shafaq Raza, Tahir Ali Chohan, Hammad Saleem

**Affiliations:** ^1^ Department of Pharmacology and Toxicology, College of Pharmacy, University of Ha’il, Haʼil, Saudi Arabia; ^2^ School of Pharmacy, Monash University Malaysia, Petaling Jaya, Selangor, Malaysia; ^3^ Institute of Pharmaceutical Sciences (IPS), University of Veterinary and Animal Sciences (UVAS), Lahore, Pakistan

**Keywords:** EGFR, anti-cancer, virtual screening, simulations, 3D-QSAR, *in-silico*, fingerprinting

## Abstract

**Introduction:**

The epidermal growth factor receptor (EGFR) presents a crucial target for combatting cancer mortality.

**Methods:**

This study employs a suite of computational techniques, including 3D-QSAR, ligand-based virtual screening, molecular docking, fingerprinting analysis, ADME, and DFT-based analyses (MESP, HOMO, LUMO), supplemented by molecular dynamics simulations and MMGB/PBSA free energy calculations, to explore the binding dynamics of quinazoline derivatives with EGFR. With strong q2 and r2 values from CoMFA and CoMSIA models, our 3D- QSAR models reliably predict EGFR inhibitors’ efficacy.

**Results and Discussion:**

Utilizing a potent model compound as a reference, an E-pharmacophore model was developed to sift through the eMolecules database, identifying 19 virtual screening hits based on ShapeTanimoto, ColourTanimoto, and TanimotoCombo scores. These hits, assessed via 3D- QSAR, showed pIC_50_ predictions consistent with experimental data. Our analyses elucidate key features essential for EGFR inhibition, reinforced by ADME studies that reveal favorable pharmacokinetic profiles for most compounds. Among the primary phytochemicals examined, potential EGFR inhibitors were identified. Detailed MD simulation analyses on three select ligands—1Q1, 2Q17, and VS1—demonstrated their stability and consistent interaction over 200 ns, with MM/GBSA values corroborating their docking scores and highlighting 1Q1 and VS1’s superior EGFR1 affinity. These results position VS1 as an especially promising lead in EGFR1 inhibitor development, contributing valuable insights towards crafting novel, effective EGFR1 inhibitors.

## Graphical Abstract

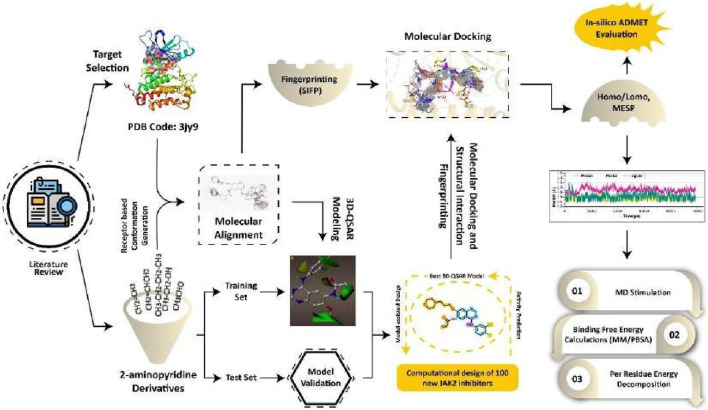

## 1 Introduction

The pressing global health challenge posed by cancer demands urgent action. In the U.S. alone, the year 2023 is expected to witness 1,958,310 new cases and 609,880 deaths. Remarkably, prostate cancer incidents have surged by 3% annually from 2014 to 2019, reversing a two-decade trend of decline. Meanwhile, lung cancer in women has been declining at a slower pace compared to men since 2015, with liver, uterine corpus, breast, and melanoma cancers witnessing an uptick in cases. This data underscores the critical need for focused research and innovative treatment strategies (Al Hrout et al., 2022; Clancy, 2023). Lung cancer is the most frequently diagnosed cancer by gender (11.6% of total cases) and the leading cause of death worldwide (18.4% of total cancer deaths), followed by breast cancer in women (11.6%), prostate cancer (7.1%), colorectal cancer (7.1%), stomach cancer (8.2%), and liver cancer (8.2%) by an 8.2% margin. Lung cancer is the leading cause of death for men and women alike. The primary cause of death among women worldwide is breast cancer, followed by lung and colorectal cancers. The diversity of cancer treatments mirrors cancer’s biological diversity. When cancer is in its earliest stages, surgical treatment is the best option. As cancer progresses, treatment typically consists of radiation, chemotherapy combinations, and, when appropriate, targeted therapies (Mohanty et al., 2019). Potential anticancer treatment based on targeted therapy. Cancer treatment side effects may be mitigated by the targeted inhibition of molecules involved in tumor development and metastasis. The signaling networks of cancer cells are responsible for their hyperactive pathways, making them a prime target for targeted treatment.

Epidermal growth factor (EGF) serves as a basis for a large family of peptide ligands that govern cancer cell growth, proliferation, and angiogenesis by binding to cell membrane receptors and activating a broad range of intracellular signalling pathways (Song et al., 2019). One of a four-membered family of transmembrane receptors, the EGF receptor (also known as ErbB1, HER1, or EGFR) is commonly overexpressed in cancer cells and is associated with a bad prognosis, just as HER2 (Nicholson et al., 2001). Consequently, EGFR is an appropriate target for the development of novel anticancer treatments. Several small molecule EGFR tyrosine kinase inhibitors are now being tested in clinical studies alongside monoclonal antibody-based therapies. The ATP- competitive selective EGFR inhibitors ZD 1839 (gefitinib, Iressa) and OSI-774 (erlotinib), which were recently authorised for the treatment of patients with advanced non-small cell lung carcinoma, are also being clinically tested in patients with other malignancies. The EGFR/HER-2 dual inhibitors quinazoline GW572016 and pyrrolopyrimidine PKI-166, as well as the irreversible inhibitors CI-1033, are all undergoing clinical assessment. *In vitro* and pre-clinical research using (PD183805) and EKB-569. ZD1839 has demonstrated remarkable antitumor efficacy. Whether used alone or in combination, against a range of malignant cells combined with different chemotherapeutic drugs. Thus, individuals who could benefit from Iressa would be identified by genetic screening before receiving medication. The fact that 80%–90% of lung cancer patients lack mutant EGFR and do not respond to currently available EGFR inhibitors motivates researchers to develop novel small molecule inhibitors that can effectively block both typical and mutant EGFR proteins (Gajiwala et al., 2013).

Therefore, the development of tyrosine kinase inhibitors has emerged as a significant topic in pharmaceutical research. However, because experimental evaluations of these compounds’ inhibitory activity are costly and time-consuming, one cannot be certain that the produced compounds always possess potent tyrosine kinase inhibitory activity. Therefore, developing a system for predicting biological activity before synthesis would be beneficial. Quantitative structure-activity relationship (QSAR) seeks information that may be used to build a model linking chemical structure to biological and other activities. Newly created compounds’ potential activity may be predicted using this method before determining whether to formally synthesise and test them. Researchers throughout the world are working to develop novel EGFR-targeting therapies as a result of treatment resistance increasing and side effects from already prescribed medications (Bhatia et al., 2020).

A comprehensive collection of 61 quinazoline derivatives with noteworthy inhibitory activity has been described recently (Smaill et al., 2016). The selectivity and potency of several of these inhibitors against EGFR1 are remarkable, therefor, using these compounds as president for the future drug design may offer novel EGFR inhibitors with pronounced anticancer properties. In this study, we used 3D-QSAR, an in-house library, molecular docking simulations, and fingerprint analysis to investigate the requirements for quinazoline and its derivatives to inhibit EGFR1. The molecular foundation of EGFR1 may be investigated by combining ligand-based QSAR with other *in silico* approaches. Strategically, 3D-QSAR contour maps were used to define the substitute properties of quinazolines derivatives. Naturally occurring quinazolines and their reported derivatives (He et al., 2017; Li et al., 2021) were used to create an in-house library of compounds. The predicted pIC_50_ values for in-house library compounds were in excellent agreement with the experimental data when using the 3D-QSAR of model compounds to assess inhibitory potential. Analysis of 3D-QSAR, docking, and fingerprinting data was able to determine the fundamental requirements and impacts associated with numerous interaction fields for extremely effective EGFR1 inhibitors. Findings from this study could aid the researcher in the rational design of powerful and selective EGFR1 inhibitors by identifying structural and pharmacophore factors guiding the binding process.

## 2 Material and methods

### 2.1 Dataset for 3D-QSAR analyses

The ligand-based 3D-QSAR models were generated based on 64 recently published quinazoline analogues which were evaluated for their inhibitory effects against EGFR1 (Smaill et al., 2016). All studied compounds were taken from the sources published by same research group at different times. The total dataset of compounds was manually divided into training and test sets according to the distribution of biological activity and chemical diversity. One-third of the total dataset was assigned to the test set for model validation, while remaining compounds were used as training set to develop 3D-QSAR model. There were 48 and 16 inhibitors in the training and test sets associated with EGFR1. Both sets cover the same range of biological activities. Those compounds which did not have biological activity for inhibition of the EGFR1 in exact numerical form were dropped from 3D-QSAR model. The IC_50_ values (nM) of studied set were converted into pIC_50_ (9- logIC_50_), which were used as dependent variables for 3D-QSAR studies (Supplementary Table S1). Each molecule was drawn with precision and then saved as a MOL2 file.

### 2.2 Compound preparation and structure alignment

The 3D-structures of the studied inhibitors were constructed by Sybyl-X1.3/SKETCH module, and then minimized by Tripos force field with GasteigereHückel atomic charge. The alignment of molecules is the second stage in creating a useful 3D-QSAR model. Since structure alignment is crucial for deducing 3D-QSAR, the process was optimized to maximize efficiency. A high-affinity molecule 1Q1 (Supplementary Table S1), was selected to be utilized as a template for database alignment. Each ligand was positioned directly on the quinazoline core ring. In the study’s subsequent analysis of the CoMFA and CoMSIA models, these alignments served as the basis for the analysis.

### 2.3 Construction of 3D-QSAR

All 3D-QSAR analysis were performed with QSAR module implemented in Sybyl-X1.3. To develop Comparative Molecular Field Analysis (CoMFA) model, the set of training ligands was aligned based on the quinazoline core of the reference compound 1Q1 and encapsulated within a three-dimensional cubic lattice, maintaining a boundary margin of 4 Å and a uniform grid spacing of 2.0 Å across all axes (X, Y, and Z). Calculation of steric and electrostatic field energies for the CoMFA descriptors was performed utilizing a probe atom with sp3 hybridization and a positive charge of +1.0. In the Comparative Molecular Similarity Indices Analysis (CoMSIA), an sp3-hybridized carbon atom, also bearing a charge of +1.0, served to assess five physicochemical characteristics associated with steric (S), electrostatic (E), hydrophobic (H), hydrogen bond donor (D), and hydrogen bond acceptor (A) interactions. The attenuation factor was maintained at the standard value of 0.3. Initial analysis employing Partial Least Squares (PLS) methodology (Cramer et al., 1988) were performed by leaveone-out (LOO) cross-validation method with default value for optimum number of components (ONC). A threshold of 2.0 kcal/mol was employed as the minimum column filtering criterion to diminish noise and enhance the efficiency of the analysis. Following this, a non-cross-validated procedure was utilized to construct the final 3D-QSAR models (CoMFA and CoMSIA), leveraging the optimal number of components (ONC) derived from cross-validation. The predictive performance of the established CoMFA and CoMSIA models was then evaluated. Compounds designated for the test set, excluded in the model development phase, were optimized and aligned following the protocol established for the training set molecules. The predictive correlation coefficient (r^2^_pred_) for these test set compounds was determined using the formula:

$$r_{pre}^{2}=\left(\frac{SD-PRESS}{SD}\right)$$ (1)

In Equation 1, SD represents the sum of squared deviations between the biological activities of the test set and the average activities of the training set molecules. PRESS denotes the sum of squared deviations between the predicted and actual activity values for each molecule in the test set.

### 2.4 Virtual screening

#### 2.4.1 Ligand-based virtual screening (LBVS)

A similarity search focusing on shape and electrostatic properties was executed through ligand-based virtual screening, employing the bioactive conformation of the inhibitor 1Q1 as the reference query. To assess the appropriateness of the chosen query for the similarity search, a validation procedure was performed utilizing the vROCS tool (OpenEye Scientific Software). Both decoy and active compounds were sourced from the Database of Useful Decoys-Enhanced (DUD-E) available at http://dude.docking.org/target/pyrd. The inclusion of decoys and actives in the validation process is pivotal for determining the efficacy of the selected query in distinguishing between known active compounds and inactive decoys in relation to the target protein [26].

The Receiver Operating Characteristic (ROC) curve, along with the Area Under the Curve (AUC) and early enrichment values, serve as the statistical metrics utilized for query validation through the vROCS program (OpenEye Scientific Software). Following successful validation, this query was employed for ligand-based virtual screening targeting chromones, chromanones, and chalcones, facilitating the execution of shape matching and electrostatic similarity assessments.

##### 2.4.1.1 Shape similarity search

Employing the validated query, the vROCS tool (OpenEye Scientific Software) conducted a search for shape similarity. The 3D conformations of the ligands, generated by the Omega program, were superimposed on the query molecule utilizing the vROCS software, which utilizes Gaussian shape overlap for scoring the ligands. Ligands were evaluated and scored according to their shape (Shape Tanimoto score) and electrostatic properties (Color Tanimoto score), subsequently being ranked based on the combined Tanimoto score (incorporating both shape and color) [26].

##### 2.4.1.2 Electrostatics similarity search

Electrostatic similarity analyses of the 3D conformers of ligands were conducted utilizing the EON software (OpenEye Scientific Software), based on the validated query. EON aligns the molecules against the query and computes the electrostatic potential using Poisson-Boltzmann and Coulombic electrostatic tools. The molecules were evaluated and scored based on the Poisson- Boltzmann electrostatics Tanimoto (ET_pb), Coulombic electrostatics Tanimoto (ET_coul), and the EON shape Tanimoto (EON_shape_tani). Subsequently, ligands were ranked based on the electrostatics Tanimoto combo (ET_combo), which integrates the EON shape Tanimoto with the Poisson-Boltzmann electrostatics Tanimoto (ET_pb) for comprehensive scoring and ranking [26].

#### 2.4.2 Docking-based virtual screening

At first, the co-crystalized structure of EGFR bonded to Lapatinib was retrieved protein data bank (PDB ID: 1XKK) (Wood et al., 2004). Before the docking process, protein structures were meticulously prepared, involving the exclusion of solvent molecules, correction of absent atoms, and geometric optimization, to preserve the structural integrity and enhance the reliability of the protein models (Stanzione et al., 2021). The Ligand-Based Virtual Screening (LBVS) identified the top 16 lead compounds utilizing metrics such as Shape Tanimoto (ST-score), Colour Tanimoto (CT-Score), and Tanimoto-Combo (TC-score). These compounds, together with the 64 ligands utilized for the 3D-QSAR model, were subjected to flexible docking into the active site of EGFR employing the Glide-XP module from Schrödinger (Friesner et al., 2004), in accordance with established protocols (Tripathi et al., 2012; Pan et al., 2013; Owoloye et al., 2022). The receptor grid was constructed using the preprocessed protein, applying the OPLS 2005 force field [25]. Adjustments to the *van der Waals* (*vdW*) radii of protein atoms were made using a scaling factor of 1.0, and a charge cutoff of 0.25 was implemented to assess polarity. The dimensions of the receptor grid box were defined as ≤ 20Å in each spatial direction (x, y, and z), centering the box around the target ligands to ensure ample space in the binding pocket for accommodating any ligand [26]. A cubic docking grid, positioned near the hinge residue M769 and tailored to enclose ligands up to ≤20Å, was generated. Glide’s extra precision (XP) scoring mechanism was employed, allowing for complete ligand flexibility during docking. The final energy assessment was conducted using GlideScore, yielding the most favorable pose for each of the 80 compounds (Hamza et al., 2023). Remarkably, the docking simulations frequently converged, indicating the lowest energy docked complex for the most similar conformations.

### 2.5 Structural interaction fingerprint (SIFt) analysis

To identify essential residues involved in ligand binding, the method of Structural Interaction Fingerprint (SIFt) analysis was utilized. This approach offers a direct method for examining the interactions between ligands and receptors, which holds considerable potential for implications in drug design and discovery. SIFt represents a strategy for the assessment of protein-ligand interactions in a three-dimensional context (Venhorst et al., 2008). The primary objective of this methodology was to create an interaction fingerprint by simplifying the intricate binding characteristics of the ligand-protein complex from three-dimensional space into a binary numeral system. This transformation allows the substantial information inherent in ligand-receptor complexes to be systematically organized, analyzed, and visualized through fingerprints, facilitating database mining activities. Utilizing SIFt as a molecular sieve in the screening process of a virtual chemical library enables the identification of compounds that exhibit desirable binding modes and interaction patterns with the protein target. The analysis conducted with SIFt focused on the three predominant contact types in ligand-protein interactions: hydrophobic contacts, hydrogen bond donors, and hydrogen bond acceptors (Deng et al., 2004). The SIFt panel within Schrödinger Release 2020-2 (2020) was employed to generate the interaction fingerprint for the protein-ligand complex. The receptor grid and ligands were chosen as the input files for this analysis. Upon generating the fingerprint, the results can be visualized in an Excel sheet format, which accentuates the residues and types of interactions that play significant roles in binding (Jasper et al., 2018). Corresponding colors were used to indicate the type of interaction shown by the residues while the numerals 1 and 0 indicated the presence and absence of interaction respectively. Typical docking research outcomes of 100 complexes were validated using fingerprint analysis (Kakarala and Jamil, 2015). Multiple binding mechanisms, variable orientations, and positions relative to the target protein were observed in the poses.

### 2.6 Toxicological modelling and ADME profiling

The information gained through ADME profiling is crucial for determining the pharmacokinetic properties of drugs, which in turn helps with decision-making, dosage selection, and the overall effectiveness of drug development program. Since it quickly reveals key insights into the absorption, distribution, metabolism, and excretion properties of drugs, ADME profiling is a time-saving catalyst in the multifaceted area of drug development. This cutting-edge methodology allows scientists to swiftly identify potential challenges and limitations, allowing them to strategically navigate the optimisation process, maximising the efficient allocation of resources, and speeding up the path towards the development of safe and effective drug compounds. The compounds’ potential for synthetic preparation was assessed using the Swiss-ADME web application (http://www.swissadme.ch). The pkCSM web application (https://biosig.lab.uq.edu.au/pkcsm/prediction), which can be accessed through the URL (https://biosig.lab.uq.edu.au/pkcsm/theory), was then used to predict the *in silico* ADMET properties of all 80 compounds (3D-QSAR model compounds and VS-hits). Swiss ADME and pkCSM tools provide rapid access to and analysis of datasets and in-house library compounds, easing the process of identifying the best candidates for future development based on their compounds’ ADME features.

### 2.7 DFT studies/MESP/HOMO/LUMO analysis

DFT calculations were conducted with minor adjustments based on the protocol previously outlined (Ejaz et al., 2022). Utilizing the Gaussian 09 software package (Revision E.01) with its standard configurations, all calculations were carried out employing the B3LYP functional alongside the SVP basis set. This theoretical framework proves effective for the determination of the electronic structures of atoms and molecules. The present investigation aims to ascertain various key parameters, including optimized geometric characteristics, the frontier molecular orbital (FMO) energies, as well as global and local reactivity indices, and the molecular electrostatic potential (MEP). The generated checkpoint files were examined using Gauss View 6.

### 2.8 Molecular dynamics simulations

The structurally modeled docking simulations of experimentally identified potent and moderate EGFR1 inhibitors, 1Q1 and 2Q17 respectively, were further refined and stabilized within a solution system through molecular dynamics simulations using the AMBER20 software package (Case et al., 2012) with the ff99SB force field. Similarly, docking complexes of compounds identified via virtual screening, as illustrated in Figure 4, bound to EGFR1, were also subjected to molecular dynamics (MD) simulations. Additionally, an evaluation of the binding affinities among the 1Q1, 2Q17, and VS1-EGFR1 complexes was conducted employing the MM/PB(GB)SA (Gohlke and Case, 2004) approach. All MD simulations, along with the molecular mechanics-based free energy estimations (MM/PB(GB)SA), were executed exclusively within the AMBER16 software environment (Case et al., 2012), according to the previously established protocols and parameters (Chohan et al., 2016a; Chohan et al., 2016b; Rehman et al., 2019). Furthermore, detailed descriptions of these methodologies are also available in the Supplementary Material.

## 3 Results and discussion

### 3.1 3D-QSAR analysis

**FIGURE 1:**
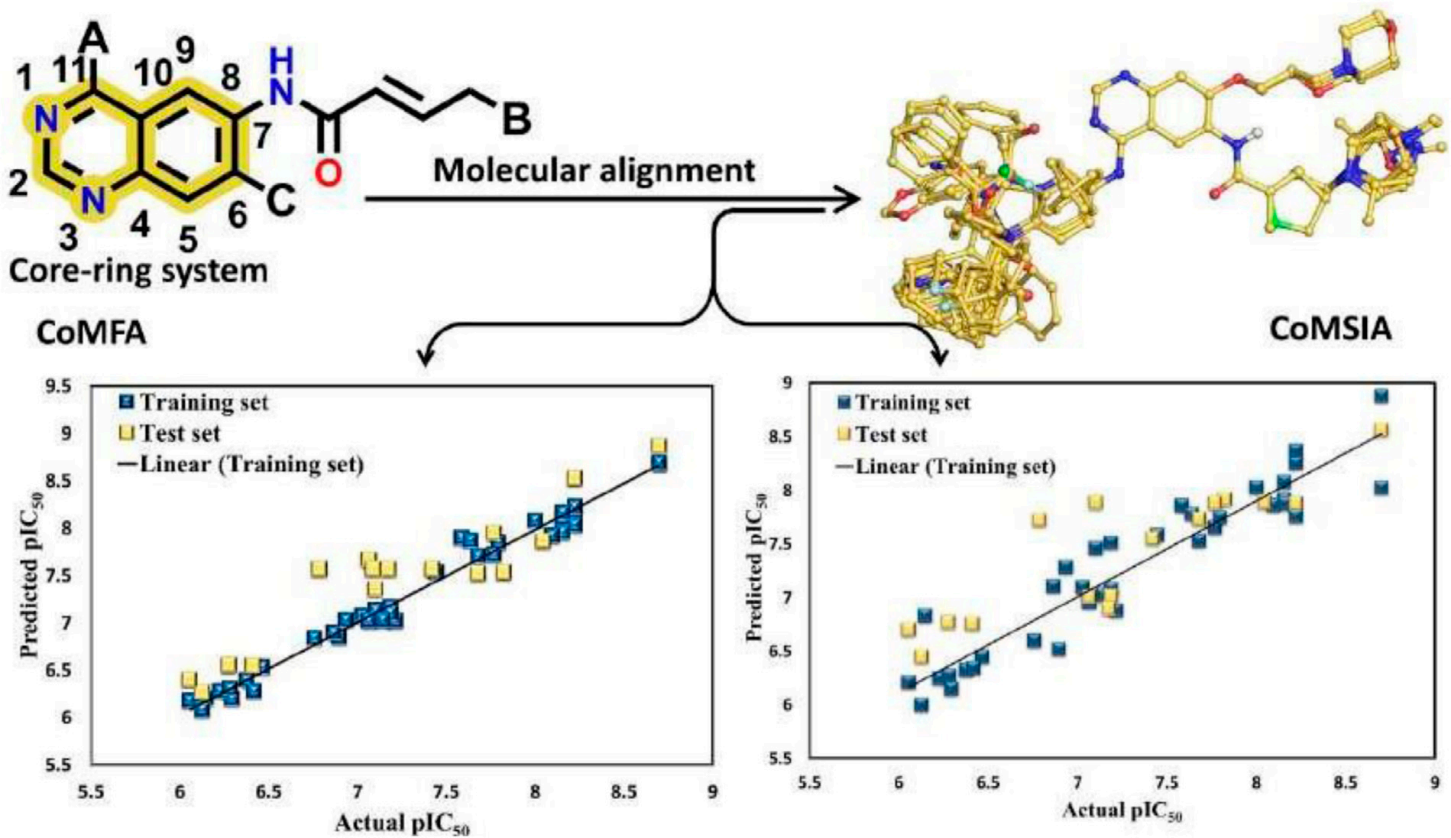
Regression analysis of pIC_50_ values predicted by the model compared to actual pIC_50_ values CoMFA values and CoMSIA values.

The 3D-QSAR model was developed using the previously reported Quniazolies analogs that were tested for their inhibitory potential against EGFR1 (Smaill et al., 2016). The molecular alignment based on the quinazoline core ring, along with the results derived from the CoMFA and CoMSIA, is depicted in Figure 1 and quantitatively detailed in Table 1. Table 1 reveals the CoMFA model’s metrics: showing q2 = 0.608, ONC = 8, Rncv2 = 0.979, SEE = 0.1126, and F = 257.401. The steric contribution (51% of the total) was found to be greater than the electrostatic effect (49%), according to the model’s steric and electrostatic fields. The CoMSIA model, assessing the QSAR model’s validity through five parameters-steric (S), electrostatic (E), hydrophobic (H), hydrogen bond donor (D), and hydrogen bond acceptor (A)-highlights the relative significance of each field. The outcomes from CoMSIA were as follows: q2 of 0.517, Rncv2 of 0.882, SEE of 0.2593, and F of 315.061, with field contributions being 23% steric, 20% hydrophobic, 21% electrostatic, and hydrogen bond donor and acceptor fields contributing 19% and 16%, respectively. These findings underscore the critical role of hydrogen bonding in ligand-protein complexes and suggest that steric and hydrophobic field contributions are beneficial for ligand binding. Figure 1 presents a scatter plot comparing observed pIC50 values against predicted pIC50 values for both the training and test sets within the CoMFA and CoMSIA analyses. The plot shows that most of the compounds align closely with the trend line, indicating a robust correlation between observed and predicted pIC50 values, thereby affirming the model’s strong predictive accuracy. This alignment signifies that predicted values are in good agreement with experimental data. The validation of the models using compounds from both the training and test sets demonstrates that the CoMFA and CoMSIA models possess substantial predictive power for assessing the inhibitory activity of compounds targeting EGFR1, as evidenced by the solid correlation coefficient values (q2 and Rncv2). This establishes their relevance in predicting the activity of EGFR1 inhibitors accurately.

**TABLE 1:** Statistical characteristics for 3D-QSAR derived from the CoMFA and CoMSIA models.

PLS statistics	CoMFA	CoMSIA
*q* ^*2*^ ^a^	0.608	0.517
*ONC* ^b^	8	5
*R*_*ncv*_^2^ ^c^	0.979	0.882
*Probability of r* ^*2*^	0.000	0.000
*SEE* ^d^	0.166	0.218
*F* ^e^	257.40	315.06
*R*_*pre*_^*2*^ ^f^		0.741
Contributions		
Steric	0.513	0.230
Electrostatic	0.487	0.214
Hydrophobic		0.201
H-bond donor		0.195
H-bond acceptor		0.160

^a^ Leave-one-out.

^b^ Optimum number of components.

^c^ Non-cross-validated correlated coefficient.

^d^ Standard error of estimation.

^e^ F-test value.

^f^ Predicted correlated coefficient.

### 3.2 CoMFA contour-map

**FIGURE 2:**
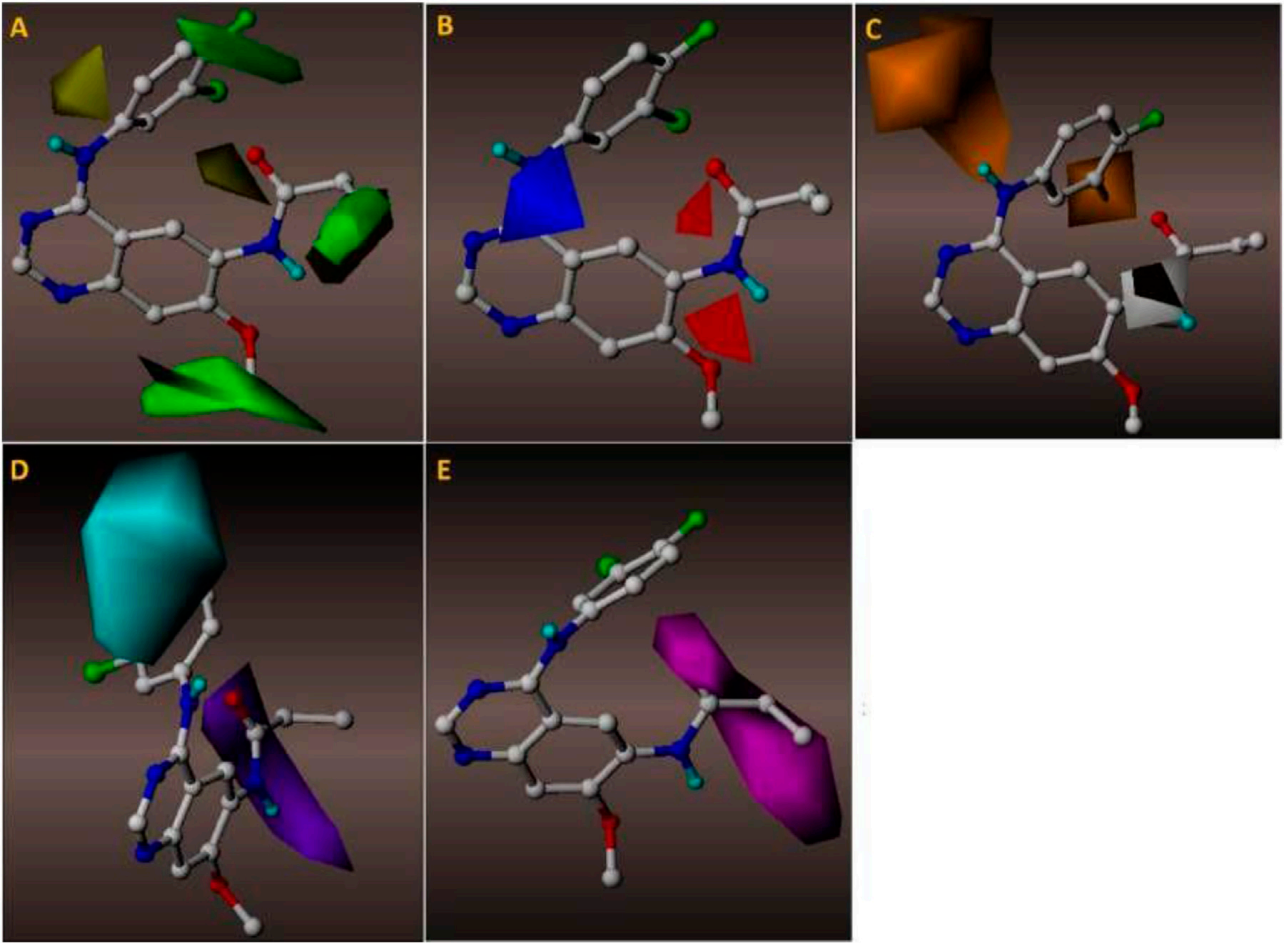
**(A)** The green contours depict the steric effect required to strategically select molecules with high activity, whereas the yellow contours discourage the bulky group. **(B)** Electrostatic contours: blue contours depict the region’s need for positively charged groups, whereas red groups indicate the presence of negatively charged groups required for adequate activity. **(C)** Hydrophobic contours: orange groups indicate activity-friendly yellow contours, whereas white contours oppose this polarity. **(D)** Hydrogen bond donor contours: purple contours signify HBD need, whereas cyan contours depict the region where HBD groups are disfavored. **(E)** Hydrogen bond acceptor contours: magenta represents a site favoured by HBA. CoMFA and CoMSIA contours illustrated in 2D.

CoMFA analysis is an effective tool in the drug discovery process. CoMFA helps find critical chemical characteristics for optimized drug design by comparing the steric and electrostatic properties of molecules with their inhibitory activities, yielding useful insights into the underlying structure-activity associations. The green contour in Figure 2A of the CoMFA analysis highlights the significance of the substitution at position 2 of the quinazoline ring. CoMFA analysis shows a solid green contour for compund 1Q1 showing best IC_50_ (Figure 2), where an oxymethyl group is immediately connected. CoMFA’s green contours indicate that a large group capable of exerting strong steric effects was needed at this location. The methylmorpholine-substituted on oxymethyl group of compound 1Q2 has a similarly impressive IC_50_ value of 7 nM. The same methylmorpoline structure, with a substitution at position 18, is seen in compound 1Q49, which has an IC_50_ value of 9 nM. Bulky group substitution is shown by the green contour at position 16 of the propionamide moiety, which is directly attached to the quinazoline ring at position 1. Compounds 1Q47 and 1Q48 highlight the significance of the first position of the quinazoline ring by associating methacrylamide and (E)-but-2-enamide chains, respectively. Both of these compounds have an IC_50_ value 21 nM and 22nM, respectively that is less than that of compound 3Q55 (in which dimethylamine is further substituted on the (E)-but-2-enamide chain). The IC_50_ for 3Q55 is just 6nM, making it a far more potent inhibitor than either 1Q47 or 1Q48. Spot 25, on the ring of the chlorofluorobenzene substituent on the amine group at Position No. 11, is the third and most crucial bulky desired group position. If this fluorobenzen ring is changed with any other bluky ring structure, the binding affinity will decrease, as seen by the yellow contour on the identical ring structure. In the instance of compound 2Q9, for example, the IC_50_ value drops by a factor of 367 when the N-methyl-1-(pyridin-3-ylmethyl)-1H-indol-5-amine ring is replaced at position 11. The yellow contour on position 13 indicates if a bulky group were to directly replace the tiny moiety with a carbonyl compound at this place, the binding affinity would decrease.

The electrostatic contribution is shown by blue and red outlines in the CoMFA analysis (Figure 2B). The area shown in blue represents the requirement for a positive charge, while the area shown in red represents the necessity for a negative one. As the red outline suggests, the existence of a negative charge at the 14 and 17 spots is critical. In the example of compound 1Q3, its high IC_50_ value is due in large part to the presence of oxygen at both of these positions. Significant positively charged compounds are required, as shown by a blue contour at position 11 on the quinazolin ring. Similar to 1Q2 and 1Q3, these compounds also have an amino group in the following positions, which contributes to their potent inhibitory effect.

### 3.3 CoMSIA contour-map

The correlation between molecular 3D structure and biological activity is studied using a subset of 3D-QSAR called CoMSIA (Comparative Molecular Similarity Indices Analysis). Incorporating new molecular characteristics, such as hydrophobicity and hydrogen bonding, CoMSIA builds on the foundation of CoMFA analysis. To determine which areas substantially contribute to the observed activity, CoMSIA computes similarity indices depending on the molecular fields around a collection of molecules. CoMSIA gives a thorough knowledge of the structural elements that impact the biological activity of compounds by taking into account the steric and electrostatic fields, as well as other variables. In Figure 2C, the orange contours indicate the position where a hydrophobic moiety is required, while the cyan colour indicates the position where the presence of hydrophobic compounds decreases the binding affinity of quinazoline derivatives with the EGFR1 target protein. Hydrophobic compounds are needed at position 11, as shown by the orange contour, and 1Q3 satisfies this criterion by virtue of the presence of an amino group. While amino groups often do not behave hydrophobically, in this circumstance they do so because they are directly attached to three atoms and possess a lone pair of electrons. The hydrophobic group is needed at position 26 of the ring substitution of fluorobenzene since this side of the molecule will interact with EGFR1’s hydrophobic cavity. The substitution of indole for this group, like that of 2Q4, is indicative of productive activity. The interaction between the compound and the ligand requires the presence of a hydrogen bond donor, represented by the purple colour at position 12 (Figure 2D). Both 2Q4 and 3Q28 contain an amino group at the location indicated by the purple hue, and both compounds depict good IC_50_ values, 6 nM and 7 nM, respectively, which are favourable. The presence of cyan contour at position 26 indicates that a bond donor is not required. In Figure 2E magenta contour at position 14 on the carbonyl ring illustrates the significance of this position as a hydrogen bond acceptor. Oxygen is present with the carbonyl group at this position. It is substantially present in all qunazoline derivatives that inhibit EGFR, indicating the significance of this position. As in the case of the compound with the most appreciable inhibitory value, 1Q3 possesses the carbonyl group as HBA at the following position. Figure 2D depicts a 2D illustration of every point to comprehend the critical positions of CoMFA and CoMSIA analysis.

### 3.4 Integration of ligand-based virtual screening with 3D-QSAR validation

The Query Model X was validated using a set of actives and decoys obtained from DUD-E (Mysinger et al., 2012). To ascertain the efficacy of the query model, metrics such as the Receiver Operating Characteristic—Area under Curve (ROC-AUC), and the Enrichment Factor (EF) were employed, as reported by vROCS. The Enrichment Factor serves as a measure of the scoring function’s effectiveness when used as a virtual screening tool against the query model, representing the ratio of the proportion of actives identified to the proportion of decoys identified, with a 95% confidence interval (95% CI). The capability of the model to enrich active compounds within the top-ranked screened database compounds is particularly emphasized. Therefore, our focus was on the Enrichment Factor at 0.5%, 1%, and 2% of the ranked database, denoted as EF 0.5%, EF 1%, and EF 2%, respectively. According to Supplementary Figure S1, there is a positive tendency towards the selection of active compounds from both active and decoy datasets, with an ROC-AUC of 0.732 ± 0.728. The Enrichment Factors were determined as EF 0.5% at 9.929 (95% CI: 0.011, 1.242), EF 1% at 0.454 (95% CI: 0.033, 1.410), and EF 2% at 0.740 (95% CI: 0.151, 1.593), as detailed in Supplementary Table S1.

Query model X was leveraged in the virtual screening of compounds sourced from the e. Molecules database, as provided by OpenEye Scientific. This repository, containing over 7.6 million compounds, utilized its 2018 version, with each molecule represented by 10 conformers, for the screening process. The screened compounds were assessed for structural similarity to the query model, based on ShapeTanimoto (ST-score), ColourTanimoto (CT-Score), and TanimotoCombo (TC-score). The top 100 leads were shortlisted, yet only the top 19 compounds, showcasing the highest ST-score, CT-Score, and TC-score, were selected for further evaluation and their potential pIC_50_ values predicted using the validated 3D-QSAR model. These leading 19 virtual screening compounds, along with their respective ST-score, CT-Score, TC-score, and both CoMFA and CoMSIA scores, are presented in Supplementary Table S3 within the Supplementary Material. The examined VS hits generally exhibited pIC_50_ values ranging between 8.5 and 6.5. Given that the least active inhibitor in our QSAR model displayed a pIC_50_ value of 6.55, VS hits with pIC_50_ values below this threshold were excluded from further consideration. Only ligands with pIC_50_ values exceeding 6.55 were included in the subsequent LBVS analysis. This LBVS process, in conjunction with 3D-QSAR predictions, facilitated the identification of the leading compound, VS1 (as shown in Supplementary Figure S1), which demonstrated a significant degree of shape and color similarity with query model X, achieving a high TC score of 1.482 with an almost equal contribution from shape and color scores. The selected top 19 VS hits also exhibited a wide range of chemical diversity, providing a diverse set of lead compounds for further retrospective analysis via molecular docking experiments. Overall, the retrospective validation results lend support to our sequential screening methodology, utilizing LBVS followed by SBVS, proving its effectiveness in distinguishing between active and decoy GPER1 ligands.

### 3.5 Structural interaction fingerprint (SIFt)

This study utilized structural interaction fingerprints (SIFt) to evaluate docking orientations of new ligands, enabling the creation of protein-specific scoring systems. Essential for “scaffold hopping” in medicinal chemistry, this method identifies ligands with distinct structures through interaction patterns, not molecular structure. SIFt maps, detailing interactions via a binary seven-bit vector for amino acids, simplified analyzing the docking results of 61 compounds from QSAR model and 19 virtual screening hits against the EGFR1. This method effectively identified inhibitors targeting critical hotspot residues in the active site, crucial for ligand-protein complex formation.

**FIGURE 3:**
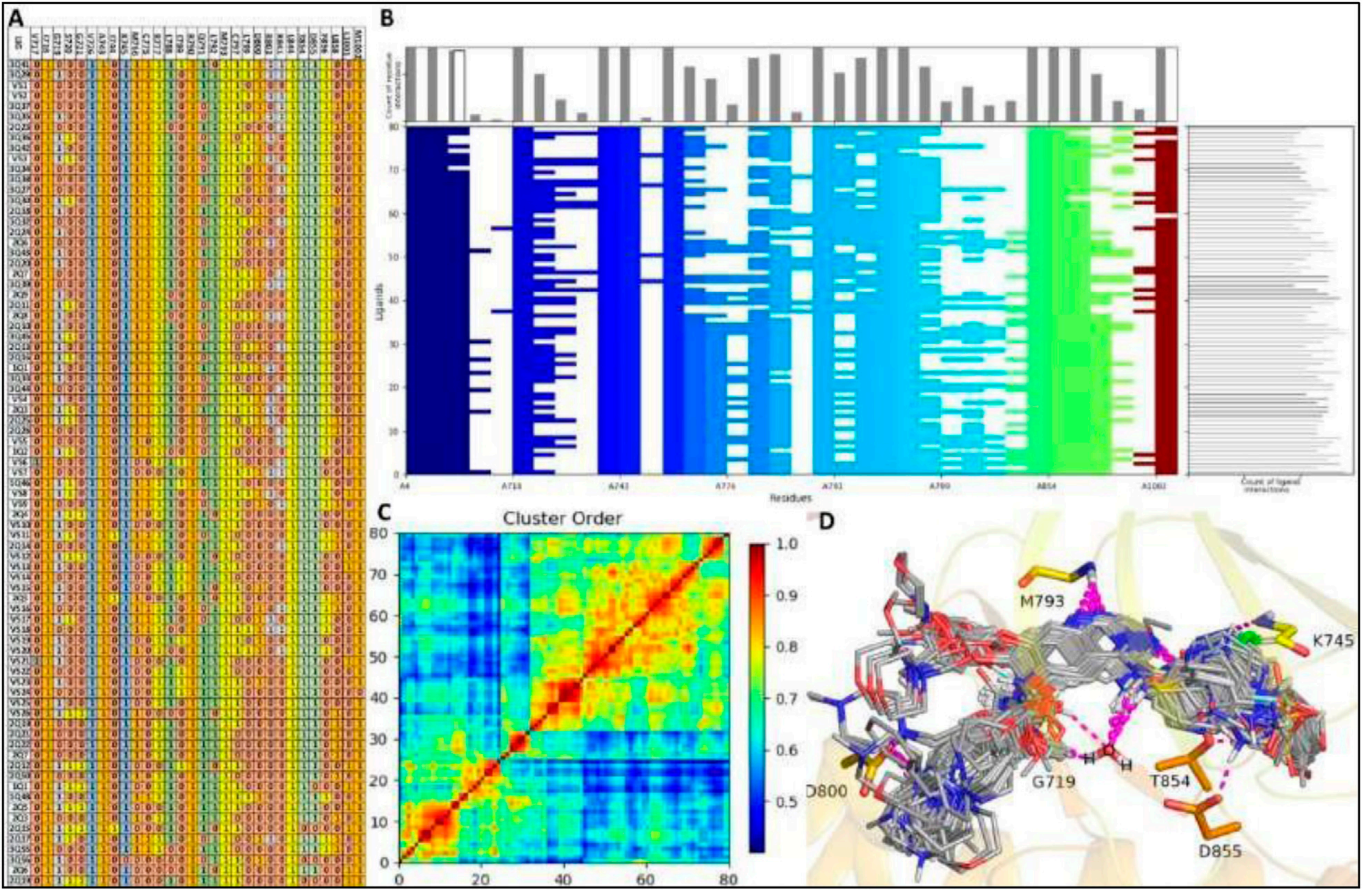
SIFt Analysis: **(A, B)** Protein-ligand interaction fingerprints for modelling compounds and VS-hits within a 4.0Å radius of interacting residues. Residue presence indicated as 1, absence as 0, and colored by hydrophobic, hydrogen bond donor, and acceptor properties. **(C)** Interaction heatmap depicting clustering analysis. **(D)** All 80 ligands (modeling and VS-hits) docked into the active site of EGFR1.

A well-constructed Structure Interaction Fingerprint (SIFt) can assist researchers in comprehending how large ligand databases bind to a protein’s active site. In this study, we developed a SIFt to analyze the binding patterns of all virtual screening hits binding to EGFR1, aiming to identify the primary hotspot residues involved in ligand-protein complex formation. Figure 3 reveals that key residues within the ligand binding cavity of EGFR1 include I718, V726, A743, K745, M769, C775, L788, R790, Q791, L792, M793, C797, D800, L844, T854, D855.

Interestingly, these same residues surround the co-crystallized ligand in 1XKK, suggesting that all ligands occupy the same binding cavity as observed in 1XKK. All the residues present in binding cavity shown interaction in fingerprinting analysis with exception of two residues V717, G721, I744, L858 of EGFR1 which only establish interaction with ligand VS6, 2Q4, VS12, VS11, and VS2, as shown in red color as hydrogen bond acceptor and sky-blue color as hydrogen bond donor in the Figure 3. Almost all ligands engage in consistent interactions with M769 and R790, making these residues pivotal in the EGFR1 binding cavity due to their essential role in these interactions. Notably, M769 and D800 participate in hydrogen bond acceptor interactions with many ligands, represented by orange in the fingerprint analysis (Figure 3). Additionally, hydrogen bond acceptor, contact, and side chain interactions occur between residue I718, V726, A743, K745, M769, C775, L788, R790, Q791, L792, M793, C797, L844, T854, D855 and ligands 1Q1 and VS1, depicted in multicolored in the fingerprint analysis. Furthermore, there is additional hydrogen bond interactions between D800 and ligands VS1, displayed in orange in the fingerprint analysis. Moreover, interactions involving hydrophobic, side chain, and π-π contacts are observed between residues A719, L764, and G772 with the majority of ligands (Figure 3). In short, our SIFt analysis indicates that not only do all the ligands exhibit high structural similarity, but they also share similar electrostatic patterns. This similarity allows the ligands to adopt analogous binding conformations within EGFR1, resulting in interaction patterns akin to those observed in the 1XKK structure. However, a few ligands, such as 2Q17, 2Q19, VS12, VS18 and VS19, exhibit slight deviations in their binding patterns compared to the co-crystallized inhibitor-EGFR1 complex. The summary of fingerprint analysis showing different colors along with ligands with highlighted regions involved in various interactions is shown in Figure 3B, which graphically depicts that about 90% or docked ligands were able to interact with 12 common residues as described previously. Hence, one may speculate these common residues as the hotspot residues for the EGFR1 inhibitors.

Figure 3C displays the heatmap generated from Schrödinger fingerprint analysis presenting a clustering of interactions among the ligands 1Q1-3Q61 and VS1-VS19 with EGFR1. The results exhibited a high degree of similarity within the 1Q1-3Q61 ligands, suggesting a conserved binding mode. Likewise, the virtual screening compounds VS1-VS19 also showed a similar range of interactions, indicating comparable binding affinities and specificities. Clusters with higher similarity scores indicated ligands with complementary steric and electrostatic features to the EGFR1 binding pocket. Some VS compounds closely mimicked the interaction patterns of high-affinity ligands, hinting at their potential as lead optimization candidates. The heatmap effectively validated the computational screening methodology and highlighted structure-activity relationship insights, essential for lead optimization. The analysis underscores promising ligand clusters for further therapeutic development, demonstrating the potential of our computational approach to identify novel EGFR1 inhibitors. The identified candidates from the VS series, particularly those analogous to the high-affinity clusters, will be prioritized for experimental validation and subsequent drug development processes.

### 3.6 Molecular docking

**FIGURE 4:**
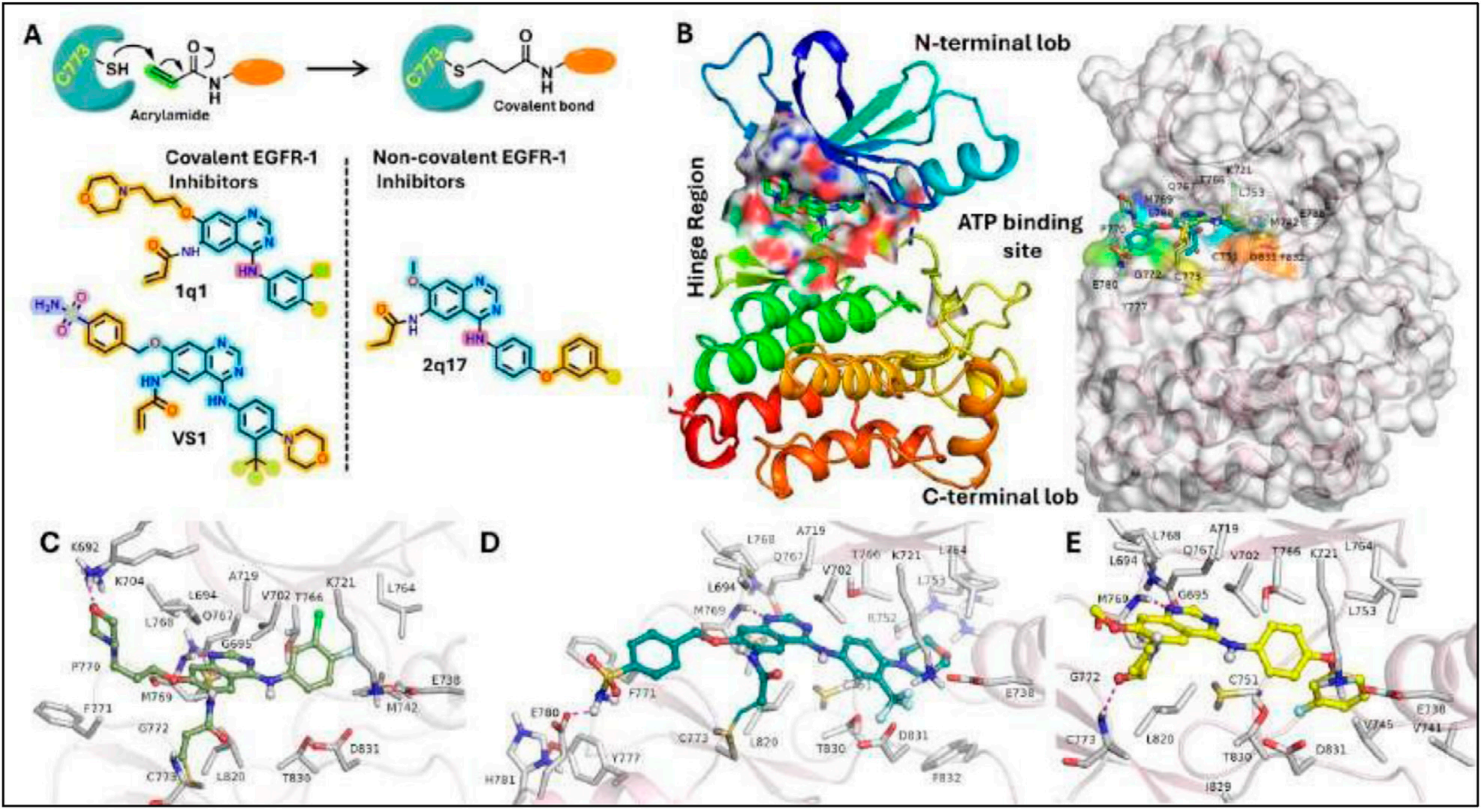
Molecular docking selected ligands into the active site of EGFR1 protein. **(A)** Schematic representation of the inhibition of target proteins (or interactions) with covalent inhibitors; **(B)** ligand binding cavity in EGFR1 protein. All selected ligands occupying the same binding site of EGFR1 protein. **(C)** Docked conformation of ligands 1Q1, 2Q17, and VS1 in their corresponding complexes **(C)** EGFR-1Q1 **(D)** EGFR-2Q17, and **(E)** EGFR-VS1.

The investigation into structural interactions elucidated by the ligand-based 3D-QSAR model was further augmented through the application of structure-based analytical techniques. Our computational model was adept at identifying essential structural molecular patterns requisite for efficacious interactions between a compound and its target protein. Docking analysis, a computational technique that assesses the interactions between small molecular compounds and target proteins, provides indispensable insights into the drug development and design paradigm. This analysis facilitates the extraction of crucial information regarding the drugs’ binding affinities, mechanisms of action, and therapeutic efficacy. To evaluate the binding efficacy of 61 model compounds and 19 hits from virtual screening against EGFR1, the bioactive conformations of all 80 compounds were computationally docked into the active site of EGFR1. The docking scores for these compounds, as detailed in Supplementary Table S2, range from–8.32 kcal/mol to −3.5 kcal/mol against the EGFR1 enzyme, indicating predominantly favorable to moderate binding affinities. Notably, the ranking of these compounds is consistent with patterns observed in experimental analyses and predictions from 3D-QSAR regarding pIC50 values. For a nuanced comparison and analysis of the binding patterns of the docked ligands within EGFR1, ligands demonstrating the highest docking scores were selected for graphical representation. According to Supplementary Table S2, compounds 1Q1 and VS1 emerge as the most efficacious model compound and virtual screening hit, respectively, achieving the highest docking scores of −8.32 kcal/mol and −8.97 kcal/mol. This suggests that VS1 exhibits a binding affinity comparable to that of 1Q1. Conversely, compound 2Q17 exhibits a moderate binding affinity (−4.46 kcal/mol) towards EGFR1. The potential binding conformations of ligands 1Q1, 2Q17, and VS1 within the EGFR1 active site are illustrated in Figures 4A–E, offering a visual understanding of their interaction dynamics. The docking analysis of EGFR1 has elucidated the distinct binding interactions of covalent versus non-covalent inhibitors. Covalent inhibitors, such as 1Q1 and VS1, form a direct bond with the cysteine residue of EGFR1, resulting in a durable modification and sustained inhibition of the target protein. This contrasts with non-covalent inhibitors like 2Q17, which do not form such bonds, suggesting covalent binding may enhance inhibitory effectiveness. Figure 4A depicts the action mechanism of covalent inhibitors, highlighting their strong, irreversible bond formation, advantageous for long-term therapeutic efficacy. Initial analysis using co-crystallized structures of EGFR1 (PDB ID: 1XKK) classified the ligand-binding pocket into distinct regions. Figure 4B shows the binding cavity’s division into hinge, solvent-exposed, and catalytic sites. Remarkably, all studied ligands occupied the ATP binding cavity in EGFR1 (Figures 3D, 4B), indicating comprehensive engagement with the target site. The structural analyses elucidate the precise positioning of covalent inhibitors within the ATP-binding site of EGFR1, highlighting a significant interaction within the hinge region, a pivotal area for the efficacy of kinase inhibitors. In contrast, the non-covalent inhibitor, 2Q17, demonstrates an alternate interaction mechanism, accentuating the superior inhibitory potential that covalent bonding offers to EGFR1. Through Figure 4A, the mechanism of action for covalent inhibitors, such as 1Q1 and VS1, is depicted, showing their engagement through a covalent bond with a cysteine residue within EGFR1-a mechanism absent in non-covalent inhibitors like 2Q17. This process of covalent bonding indicates a robust and irreversible inhibition, offering significant therapeutic benefits for sustained activity. To further dissect the ligand-binding domain of EGFR1 into specific zones, characterized by their intrinsic properties, an initial investigation utilized co-crystallized structures of EGFR1 (PDB ID: 1XKK). Figure 4B delineates the EGFR1 protein’s binding cavity, strategically located between the N- and C-terminal lobes, which can be partitioned into the hinge region, solvent-exposed region, and catalytic site. Notably, all ligands under examination were found to occupy the ATP binding cavity in EGFR1, as shown in Figures 3D, 4B. This observation underscores the extensive and intricate engagement of these compounds with the target site, demonstrating the nuanced interplay between structural features and inhibitory function within the realm of EGFR1 interactions.

To elucidate the variance in docking scores attributed to diverse interaction patterns, the optimal docking conformations for each compound were thoroughly documented and visually represented (Figures 4C–E). The binding sites for the ligands 1Q1, 2Q17, and VS1 within the EGFR1 protein are illustrated in Figure 4B, revealing that all three ligands share the same binding site. The graphical analysis demonstrates that the EGFR1-ligand complexes engage common residues I718, V726, A743, K745, M769, C775, L788, R790, Q791, L792, M793, C797, L844, T854, D855 within the ligand binding site (Figure 2A). Furthermore, validation of our docking methodology indicates a similar interaction pattern among the ligands 1Q1, 2Q17, and VS1 with the pivotal residue M769 in the hinge region, where they are capable of forming at least one hydrogen bond (Figures 4C, D). However, a significant distinction emerges due to the presence of a “warhead” (a reactive functional group) in ligands 1Q1 and VS1, enabling them to form a covalent bond with the adjacent residue C773, an interaction absent in 2Q17. In the bonded system of VS1, the—SO2NH2 group extends towards the solvent-exposed region, donating a hydrogen bond to D800. Meanwhile, the pyran moiety in 1Q1 projects outward to form a hydrogen bond with K692, and notably, 2Q17 also establishes a hydrogen bond with C773. The larger structures of 1Q1 and VS1 cover more extensive areas within EGFR1, facilitating additional interactions not seen with 2Q17. These findings significantly advance our comprehension of ligand-EGFR1 interactions and pave the way for the design of novel inhibitors with enhanced selectivity and efficacy. The data presented in this study are pivotal for the development of targeted therapies against diseases implicated with EGFR1. Further experimental validation and characterization of these findings would reinforce their significance and contribute to advancing these compounds through the drug development process.

### 3.7 Toxicological modelling and ADME profiling

The comprehensive analysis, detailed in Supplementary Table S3, represents the ADME properties, physicochemical characteristics, pharmacological toxicity, mutagenesis profile, and synthetic accessibility for the compounds evaluated in our study. This data is pivotal, providing a foundation for the advancement of these compounds through the drug development pipeline. Synthetic The ease of synthesis, quantified through synthetic accessibility scores, highlights the practicality of compound development, with most compounds achieving scores of 5 or below, suggesting feasible synthesis. Notably, the synthetic accessibility score of 2Q17 stands at 3.91, indicating a more straightforward synthesis compared to 1Q3, which has a score of 2.68. This discrepancy can be attributed to the smaller molecular structure of 1Q3. Additionally, VS1- VS4 emerges as the most promising molecule within our in-house library, with a synthetic accessibility score of 3, underscoring our library’s potential for yielding readily synthesizable compounds. The absorption rates, particularly intestinal absorption, significantly influence a drug’s bioavailability. Our findings reveal high absorption rates across the board, with 1Q3 demonstrating an absorption coefficient of 90%, indicative of excellent bioavailability. Furthermore, the intestinal absorption of the in-house compound W59 surpasses that of 1Q3, suggesting even higher bioavailability. The metabolism of these compounds predominantly involves cytochrome P450 enzymes, especially 2D6 and 3A4, essential for the metabolic breakdown and clearance of drugs. The total clearance rates, a measure of the drug’s elimination from the body, are crucial for determining appropriate dosing to achieve steady-state concentrations. Our study provides these rates in terms of log (mL/min/kg), aiding in the optimization of therapeutic dosages. Importantly, the AMES toxicity test results reveal most compounds, including 1Q3 and W59, to be non-mutagenic, suggesting a low risk of genotoxicity and a favorable safety profile for further development. Furthermore, Q1 and 2Q17 show impressive intestinal absorption rates of 90.396% and 92.87%, respectively, highlighting their potential for effective gastrointestinal uptake. VS1 stands out with an absorption rate of 99.812%, indicating exceptional bioavailability. The metabolism profiles of these compounds suggest interactions with CYP3A4, indicating their metabolic pathway involves this significant enzyme, which could influence drug-drug interactions. The total clearance rates suggest efficient excretion without rapid elimination, beneficial for sustained action. All three compounds are characterized as non-mutagenic, suggesting a promising safety profile. Present analysis, employing tools like the pkCSM web application, has facilitated a detailed evaluation of these compounds, showcasing their therapeutic potential. The highlighted absorption rates, manageable metabolism, and non-toxic nature of compounds such as 1Q1, 2Q17, and VS1 position them as promising candidates for further drug development. Their synthetic accessibility supports the feasibility of their production and development into therapeutic agents. Given their distinct interaction patterns with critical metabolic enzymes, a tailored approach to drug development might be advantageous to exploit their full therapeutic potential. Findings of ADME analysis not only enrich our understanding of the pharmacokinetic properties of the evaluated compounds but also delineate clear pathways for their optimization as therapeutic agents. The detailed ADMET profiles, particularly for 1Q1, 2Q17, and VS1, alongside the broader insights provided by our comprehensive dataset, will be instrumental in guiding the subsequent phases of drug development, including formulation, dosing, and clinical trials.

### 3.8 DFT and MESP studies

**FIGURE 5:**
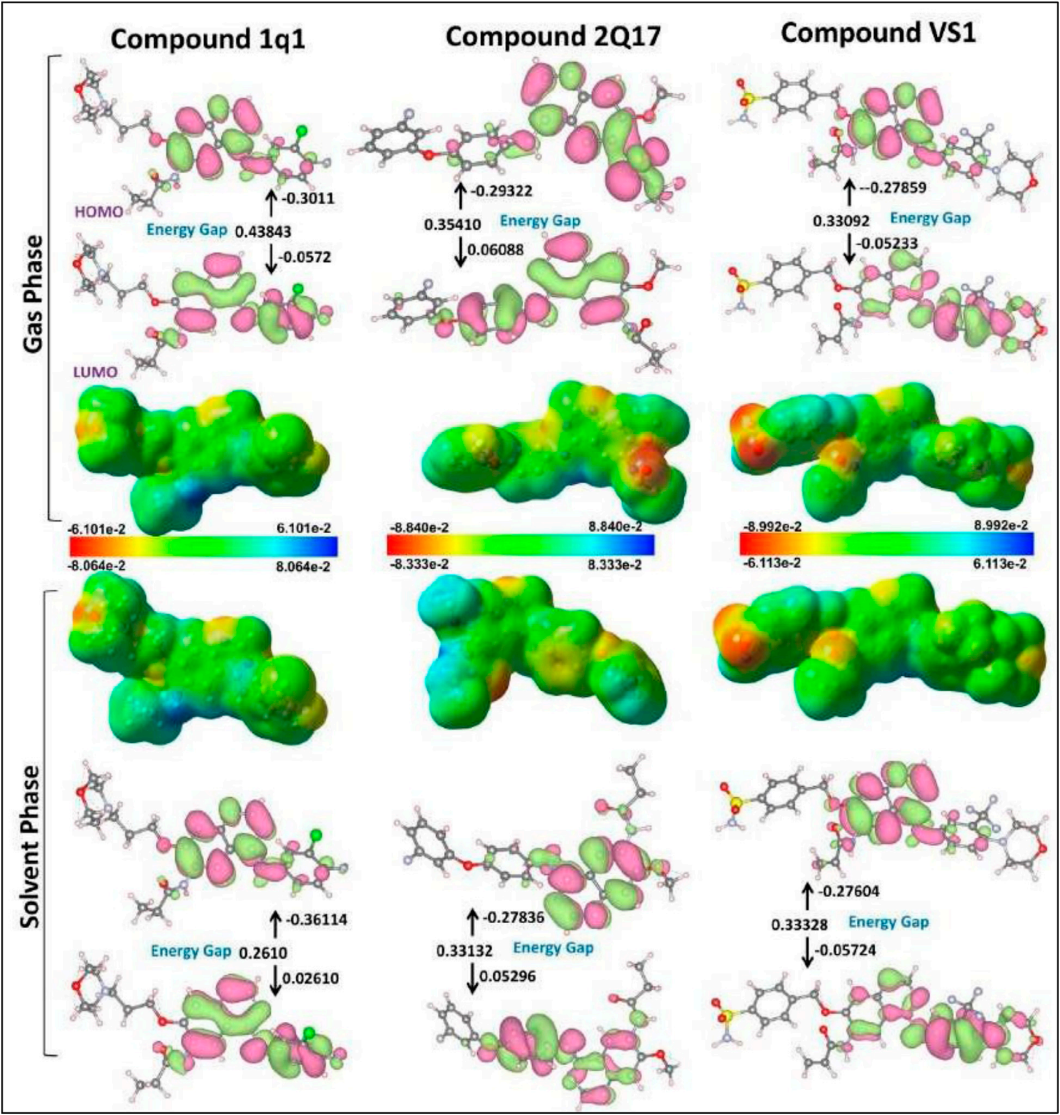
ESP structures (in both gas and solvent phases) formed by mapping of total density over electrostatic potential, and optimized structures 1Q1, 2Q17 and VS1. Calculated HOMO and LUMO orbitals of potent derivatives at B3LYP/SVP level of DFT calculations for all selected ligands.

The MESP (Molecular Electrostatic Potential) mappings have provided a comparative view of the electronic characteristics inherent to the EGFR1 co-crystallized inhibitor 1Q1 and the VS-hit VS1, distinguishing them from the moderately active EGFR1 inhibitor 2Q17. These insights, depicted in Figures 4, 5, elucidate the unique electronic properties that confer distinct biochemical interactions with EGFR1. The MESP mappings highlight the most electronegative potential regions—signified by a deep red color—on the pyran and -SO2NH2 groups in 1Q1 and VS1, respectively. These regions are pivotal, indicating areas favorable for electrophilic attack, which is critical for binding efficacy. Further detailed examination through Mulliken population analysis reveals that the nitrile oxygen atoms within the pyran ring of 1Q1 possess an average Mulliken charge of −0.85, marking the most negatively charged area surrounding the molecule. Another area of interest in 1Q1 is a relatively less pronounced negatively charged region found over the common nitrogen atom of its quinazoline core, which carries a Mulliken charge of −0.612. These negatively charged areas of 1Q1 are implicated in forming hydrogen bonds within both the hinge and the solvent-exposed regions of EGFR1, as corroborated by our docking results (Figure 3). Similarly, the quinazoline nitrogen atoms in both VS1 and 2Q17 exhibit negative charges and participate in forming common hydrogen bond interactions within the hinge region of EGFR1. Notably, like 1Q1, VS1 also harbors a negatively charged -SO2NH2 moiety, extending towards the solvent-exposed region to form a hydrogen bond with D800. In contrast, 2Q17 lacks a negatively charged moiety capable of forming hydrogen bonds in the solvent-exposed region, which may explain its reduced activity compared to 1Q1 and VS1. These findings underscore the importance of specific electronic properties and their contributions to the binding interactions between the inhibitors and EGFR1. The presence of negatively charged regions in 1Q1 and VS1, facilitating crucial hydrogen bond formation, highlights their superior inhibitory potential. Conversely, the absence of such features in 2Q17 limits its interaction capabilities, underscoring the significance of detailed electronic analysis in understanding and predicting the behavior of potential inhibitors. This analysis not only enhances our understanding of the molecular interactions at play but also opens avenues for designing novel inhibitors with optimized binding characteristics.

Frontier molecular orbital analysis can be utilized to determine the reactivity of a compound. The kinetic stability of the molecule is quantified through the HOMO-LUMO energy gap, denoting the energy disparity between the HOMO and LUMO states. Notwithstanding the compound’s high chemical reactivity, enhanced energy transmission within the molecule is enabled by the HOMO LUMO gap (Roney et al., 2023). The initial molecular orbitals to participate in a chemical reaction are the lowest unoccupied molecular orbital (LUMO) and the highest occupied molecular orbital (HOMO); these orbitals are crucial for determining the chemical reactivity of molecules. Figure 5 illustrates the molecular surface plotting of HOMO and LUMO frontier orbitals for compounds CPZ, CP1, and CP2. In receptor–ligand complex formation, the electron acceptor ability of an inhibitor molecule is dictated by the frontier orbital LUMO value, while the electron-donating ability is directly linked to the HOMO value. Table 2 provides a comprehensive summary of the computed quantum chemical descriptors, encompassing HOMO and LUMO values for CPZ, CP1, and CP2 in both gas and solvation (water) phases. The DFT calculations have elucidated critical molecular properties of the EGFR1 inhibitors 1Q1, 2Q17, and VS1, revealing their electronic structures and reactivities in both gas and aqueous phases. Notably, the dipole moment, HOMO-LUMO energies, energy gaps, and other reactivity descriptors such as ionization potential, electron affinity, electronegativity, hardness, softness, and electrophilicity were thoroughly analysed. The analysis indicates that 1Q1 exhibits a significant increase in dipole moment when transitioning from gas to aqueous phase, from 6.3580 to 8.0173 Debye, suggesting an enhanced interaction capability with the EGFR1 active site in a physiological environment. This increase in dipole moment, coupled with a decrease in the energy gap from 0.43843 to 0.35843, signifies a higher reactivity in aqueous conditions, which is favourable for effective inhibition. Furthermore, ionization potential of 1Q1 decreases from 9.56 eV in the gas phase to 8.19 eV in the aqueous phase, while its electron affinity improves, indicating a greater tendency to donate electrons and form stable interactions with EGFR1. The corresponding changes in electronegativity, electrochemical potential, and hardness suggest a balanced reactivity profile, making 1Q1 an effective EGFR1 inhibitor. In comparison, assuming similar analyses were conducted for 2Q17 and VS1, we could expect that differences in their dipole moments, HOMO- LUMO gaps, and other molecular properties directly correlate with their inhibitory activities against EGFR1. For instance, VS1’s notably high dipole moment in both phases may correlate with its exceptional EGFR1 inhibitory properties, as it indicates a strong electrostatic interaction potential. Conversely, if 2Q17 exhibited higher energy gaps and lower dipole moments compared to 1Q1 and VS1, this could explain its weaker binding affinity and inhibitory activity, as it would suggest lower reactivity and interaction capability with the receptor. These insights not only provide a deeper understanding of the molecular basis for the inhibitory activity of these ligands but also highlight the importance of optimizing electronic and structural properties for the development of potent EGFR1 inhibitors. The detailed electronic structure analysis serves as a foundation for further structure-activity relationship studies, guiding the design of new inhibitors with enhanced therapeutic profiles.

**TABLE 2:** DFT calculation (Quantum chemical descriptors) of the selected ligands.

Ligand	Dipole moment (debye)	HOMO (a.u.)	LUMO (a.u.)	Energy gap (ΔE_Gap_)	Ionization potential (eV)	Electron affinity (eV	Electronegativity χ (eV)	Electrochemical potential μ (eV)	Hardness η (eV)	Softness S (eV)	Electrophilicity ω (eV)
1Q1 Gas	6.3580	−0.3011	0.0572	0.4384	9.56	−2.38	3.59	−3.59	11.93	0.084	0.54
1Q1 Aqueous	8.0173	−0.3111	0.0261	0.3584	8.19	−1.56	3.32	−3.32	9.75	0.10	0.56
2Q17 Gas	6.3278	−0.2932	0.0608	0.3541	7.98	−1.66	3.16	−3.16	9.64	0.104	0.519
2Q17 Aqueous	9.1002	−0.2783	0.0529	0.3313	7.57	−1.44	3.07	−3.07	9.02	0.111	0.522
VS1 Gas	10.9010	−0.2785	0.0523	0.3309	7.58	−1.42	3.08	−3.08	9.00	0.191	0.526
VS1 Aqueous	14.374	−0.2760	0.0572	0.3332	7.51	−1.56	2.98	−2.98	9.07	0.110	0.489

### 3.9 Dynamic simulations, comprehensive analysis of structural flexibility and stability

In our current work, molecular dynamics (MD) simulations alongside free energy calculations were conducted to explore the interaction modes and binding mechanisms of inhibitors with varying affinities towards EGFR1. Notably, compound 1Q1 demonstrates approximately a 420- fold enhanced inhibitory potential against EGFR1 compared to 2Q17, as depicted in Figure 4. A detailed analysis comparing 1Q1 with the moderately active compound 2Q17, highlighted in Figure 4A, reveals three structural modifications that significantly boost the EGFR1 binding efficiency of 1Q1 over 2Q17. These changes include the substitution of the methoxy group on the quinazoline ring with a 4-butylmorpholine chain, the introduction of a but-3-en-2-one warhead in place of butan-2-one, and the replacement of a meta-substituted 1-chloro-2-(3-fluorophenoxy) benzene group with a 1-chloro-2-fluorobenzene group at the para position. Moreover, compound VS1, which shares a similar structure and EGFR1 inhibitory potency with 1Q1, as shown in Figure 4A, further emphasizes the structural basis for enhanced activity. Docking studies reveal that all three inhibitors achieve a remarkably similar “V”-shaped conformation within the ATP binding pocket of EGFR1, as illustrated in Figures 4C–E. Consequently, 1Q1, 2Q17, and VS1 were selected for MD simulation and binding free energy analysis to pinpoint the critical structural elements required for EGFR1 selectivity, laying the groundwork for the development of potent EGFR1 inhibitors.

**FIGURE 6:**
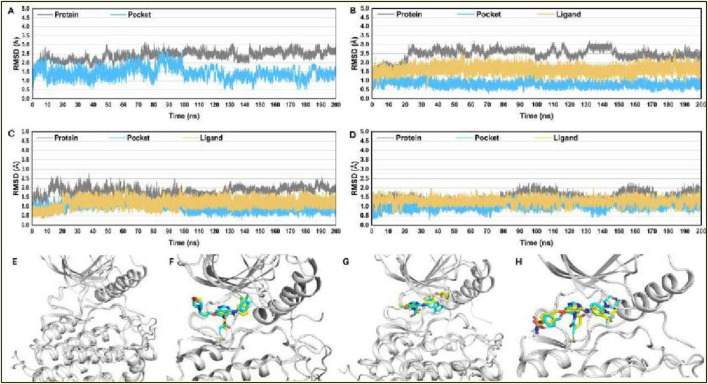
RMSD plots for studied complexes **(A)** EGFR1-APO, **(B)** EGFR1-1Q1, **(C)** EGFR1- 2Q17, and **(D)** EGFR1-VS1 after 200 ns. Post-simulated snapshots from MD trajectories of selected complexes are superposed over co-crystalized structures corresponding complexes **(E)** EGFR1-APO, **(F)** EGFR1-1Q1, **(G)** EGFR1-2Q17, and **(H)** EGFR1-VS1.

To assess the dynamic stability of our systems and verify the sampling technique’s validity, we monitored the root-mean-square deviation (RMSD) relative to the starting structures over 200 ns of molecular dynamics (MD) trajectories. The RMSD analysis confirmed that all the studied systems, including APO-EGFR1 and its complexes with the inhibitors, reached equilibrium within the first 5 ns. Notably, RMSD values for the protein’s Cα atoms, the binding pocket’s backbone atoms, and the ligands’ heavy atoms after reaching equilibrium averaged approximately 2.5 Å, 1.6 Å, and 1.2 Å, respectively. These findings, as illustrated in Figures 6A–D, demonstrate the systems’ stability, providing a solid basis for subsequent hydrogen bonding free energy and energy decomposition analysis using conformations sampled from 5 to 100 ns. The stability of these conformations was further validated by overlaying the coordinates of representative MD-simulated snapshots over their initial conformations, as shown in Figures 6E–H. This structural analysis revealed that all complexes and APO-EGFR1 maintained stability throughout the simulation, with all ligands preserving their initial conformation and essential hydrogen bonds with the hinge residue M769. These results underscore the reliability of our MD simulation outcomes for further binding free energy analysis, offering promising insights into the detailed interaction mechanisms of these inhibitors with EGFR1.

**FIGURE 7:**
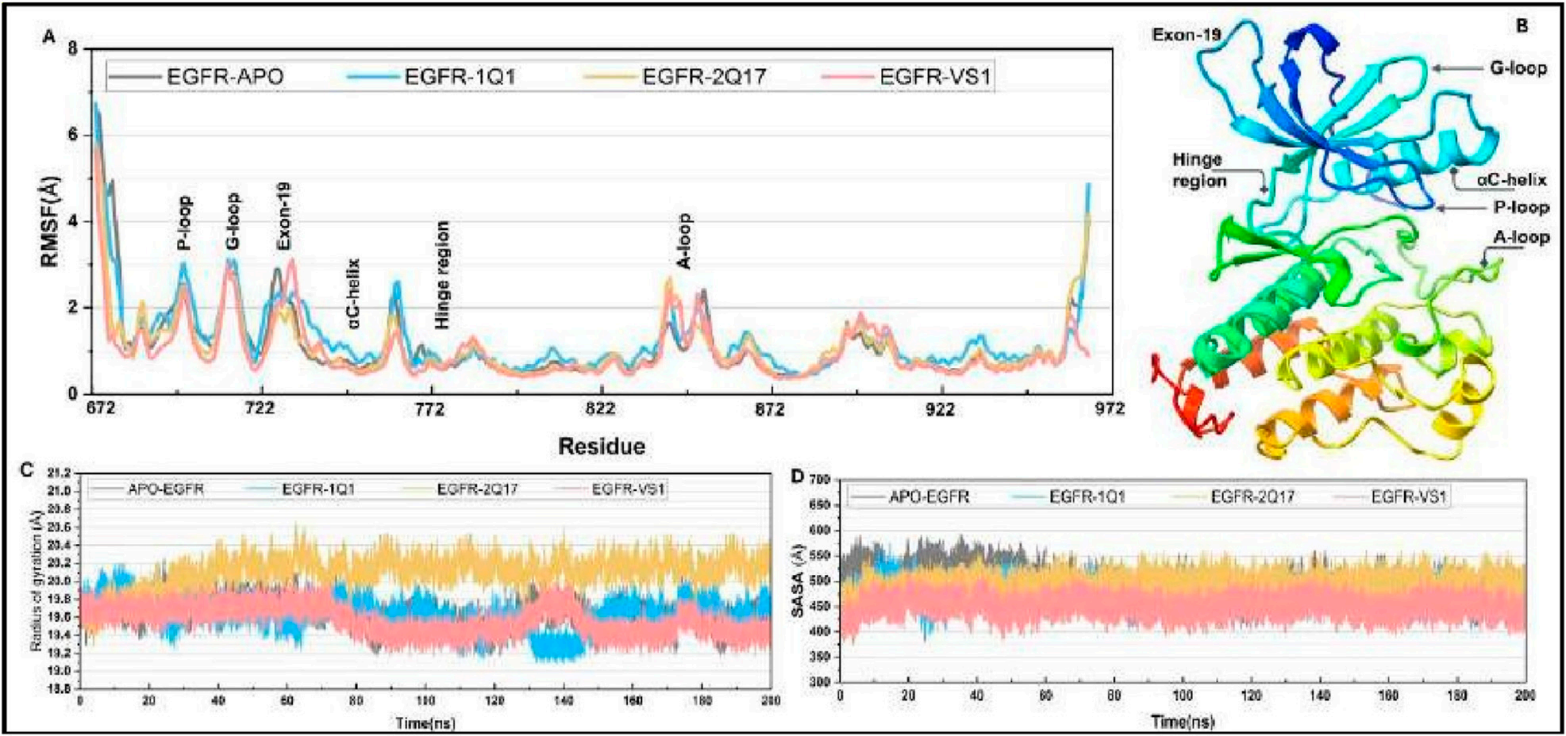
**(A)** Root mean fluctuation (RMSF) curve for selected complexes and their comparison to APO-EGFR1 structure. **(C, D)** Plots for radius of gyration (ROG) and solvent accessible surface area (SASA) for all complexes.

Additionally, a root-mean-square fluctuation (RMSF) analysis was conducted for all ligand-protein systems, as showcased in Figure 7A. This analysis revealed that the dynamic characteristics and RMSF distributions across the protein structures of all systems followed similar patterns. Notably, six regions within the EGFR1 structure-the P-loop, G-loop, Exon-19, α-helix (adjacent to the hinge region), the hinge region itself, and the A-loop—exhibited the most pronounced fluctuations, as detailed in Figure 7B. Specifically, the G-loop region (residues 710–722) displayed comparable fluctuations in both nonbonded and bonded systems with EGFR1, while the Exon-19 region exhibited slightly enhanced fluctuations in systems bonded with EGFR1-VS1. This suggests that ligand binding enhances the mobility of the Exon-19 and A-loop within EGFR1. Although the G-loop and P-loop showed similar fluctuation patterns across the board, EGFR1- VS1 and 1Q1 complexes experienced heightened fluctuations in these loops. Conversely, the α-helix and hinge region demonstrated stability in all three EGFR1 systems, indicating their crucial role in maintaining the helix’s stability upon ligand binding. The A-loop residues, when part of the receptor-ligand bonded systems, showed a greater fluctuation amplitude compared to those in the unbound EGFR1, emphasizing the dynamic impact of ligand association on this region’s mobility.

To assess the influence of ligand association on the structural compactness of the protein, the radius of gyration (Rg) for EGFR1 was monitored over the duration of the simulation, as illustrated in Figure 7C. The computed average Rg values for EGFR1 when complexed with 1Q1, 2Q17, and VS1 were approximately 20.1 Å and 19.8 Å and 19.9, respectively, showcasing a remarkable uniformity. Such consistency underscores a negligible impact on the protein’s overall conformation in response to ligand interaction, indicating that ligands understudy engage with the enzyme without prompting significant structural alterations like unfolding or expansion. Moreover, the stability of Rg values within the tight range of 19 Å to 20Å throughout the simulation period underscores the substantial structural integrity maintained. These results collectively imply that the interaction of either 1Q1, 2Q17 and VS1 with the protein does not perturb its native conformation, which may play a pivotal role in their mechanism of inhibition by preserving the architecture of the active site essential for effective ligand recognition.

Furthermore, an analysis of the solvent-accessible surface area (SASA) was conducted to further elucidate the dynamics between EGFR1 and the investigated inhibitors (Figure 7D). The SASA value observed for the complex bound with 1Q1 was approximately 540 Å, indicating a stable interaction within this complex. Intriguingly, 2Q17 demonstrated a stability on par with acarbose, as reflected by their comparable average SASA values, approximately around 500 Å. Moreover, the VS1 also displayed similar range of SASA value 450 Å. Whereas the highest SASA value was observed in case of APO-EGFR1 proteins (Figure 7D). This parallel in stability reflects the potential efficacy of studied ligands as potential inhibitor of EGFR1.

#### 3.9.1 Principal component analysis (PCA) and free energy landscape (FEL) of the EGFR1- 1Q1 complex

**FIGURE 8:**
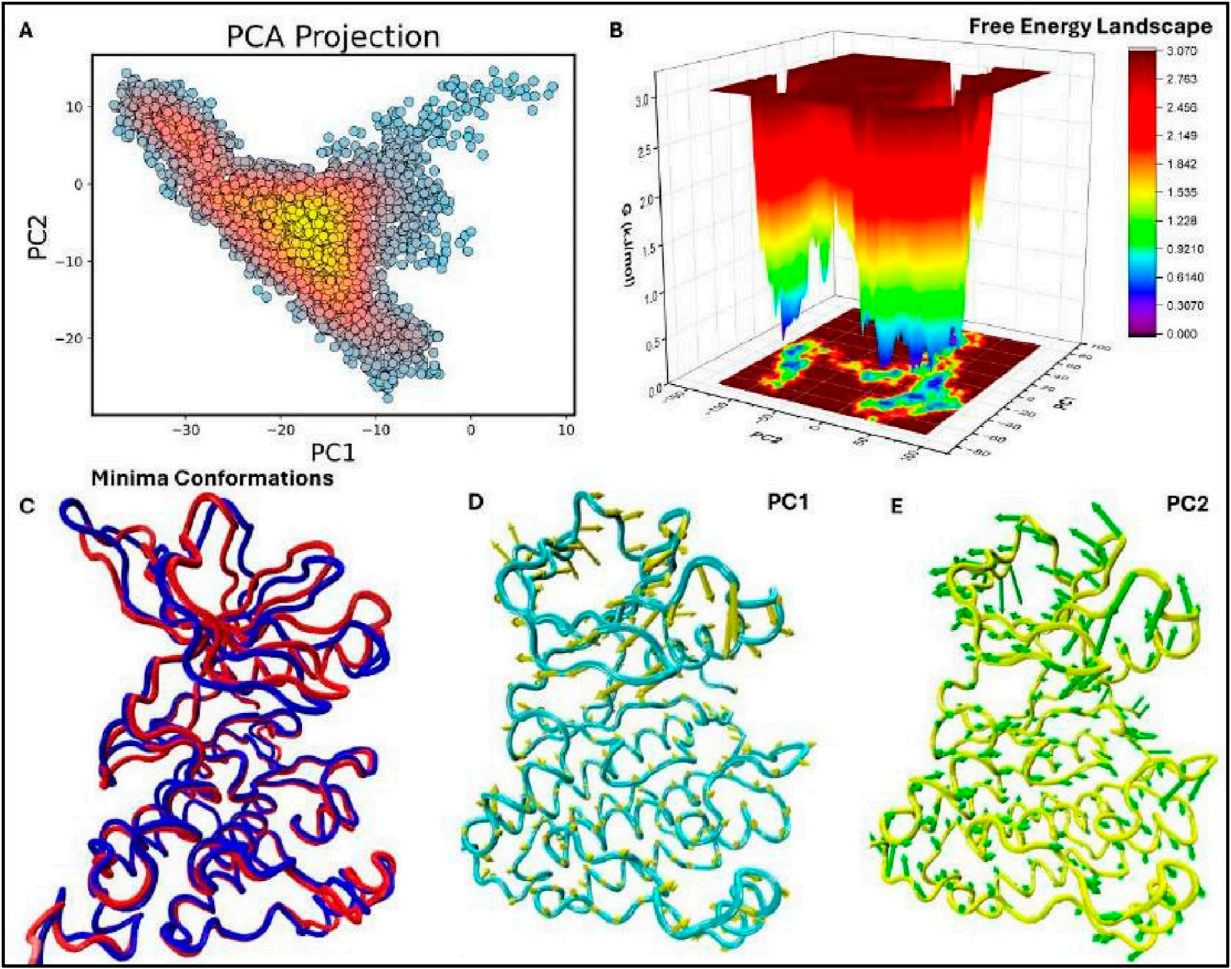
Figure: Principal Component Analysis (PCA), Free Energy Landscape (FEL), and Porcupine plots of the EGFR1 system bound to the most potent inhibitor, 1Q1. **(A)** The top-ranked principal components (PC1, PC2) from the last 50 ns of MD trajectories are plotted against each other. **(B)** 3D FEL plot of the 1Q1-EGFR1 complex, illustrating the free energy distribution between PC1 (*x*-axis) and PC2 (*y*-axis). The color gradient indicates Gibbs free energy, ranging from the lowest energy conformations (blue) to the highest (red). **(C)** Superimposition of the FEL minima, highlighting conformational changes between the lowest energy states. Minima I and II are shown in blue and red, respectively. **(D, E)** Porcupine plots representing extreme PC1 and PC2 projections for all simulated systems. The direction and length of the yellow (PC1) and green (PC2) arrows at each Cɑ atom illustrate the direction and magnitude of motion. The protein is displayed in tube form, with regions of significant fluctuation labeled.

In the EGFR1 protein complexed with the 1Q1 ligand using Principal Component Analysis (PCA) the structure of the protein undergoes considerable conformational change throughout the time period of the simulation. As observed from the scatter plot of the first two principal components (PC1,PC2) (see Figure 8A), majority of the conformations are clustered in the low energy basins which are represented by yellow and red regions. These regions are representative dynamic behavior, presumably the most physiologically relevant conformations of EGFR1 with explores a wide kinetic space, although the dominant stationary points are responsible for the binding. The distribution observed parallel with both principal axes indicates that the system of what are essentially quite stable conformations that are required for proper protein-ligand the 1Q1 ligand.

To similarly assess the stability of the identified conformations, the previously generated FEL was overlaid along the PCA projections (refer to Figure 8B). The FEL defines two distinct target locations that are consistent with the two lowest forces with respect to the complex between EGFR1 and the 1Q1 ligand. Potential energy is plotted against conformation for two different proteins with an energy minimum shown in blue that suggests an affinity of the system for certain conformations requiring minimal amount of energy. These steady states are expected to improve the extent to which 1Q1 inhibits EGFR1 by stabilizing regions of the protein that are crucial so as zones of the protein. These changes suggest that even though the overall structure of the EGFR1 I (blue) and II (red) demonstrate minor but essential conformational changes mainly in the flexible rationale has been provided concerning the structural changes of these stable states. Here, Minima occurring between superimposition of the two lowest energy minima conformations (Figure 8C), a to prevent ungainly rearrangements that lead to receptor activation. In identifying the changes is well preserved, the presence of the inhibitor influences small motions that could affect the dynamics of the receptor. This flexibility is important for the biologic activity of EGFR1 because it allows the extant polypeptide to interconvert to other conformations required for ligand recognition or activation.

To express characteristics of dynamics of EGFR1-1Q1 system, porcupine plots are shown in observation compares well with the previously proposed model that 1Q1 immobilizes critical flexibility in certain areas of the protein for maintenance of the functional motions. This changes mostly occur in the areas distal to the ligand-binding site, underlining the roles of time series larger arrows stand for larger change in the position of the atoms. Importantly, these plots, arrows also show direction of the changes in the position of Cα atoms and the signifying of Figures 8D, E, which demonstrate the motions in the principally first two components. In these regions of EGFR1 to provide stability necessary for structural rearrangements while allowing the protein enough wiggle room to enable ligand binding or allosteric regulation. The PCA and FEL together, the present results enable a systematic appreciation of the conformational disposition of conformation alteration which could in part be the ligand activation or inhibition process. Taken for extended periods the architecture is fairly constant with local flexibility important in allowing need for stability and the need for flexibility within the structure of a large and successful complex. Transduction which makes it function inhibitory. These porcupine plots also firmly establish the conformations, 1Q1 may not allow EGFR1 to adopt active conformational states needed for signal particular conformations to act as an inhibitor. By engaging the protein into energetically favorable stability and function. The 1Q1 ligand’s lock-and-key property is owing to the stabilisation of these analyses support that the EGFR1-1Q1 complex is in low energy conformational states required for EGFR1-1Q1 and the dynamic profile of the system that will be use for designing enhanced inhibitors. Possible future studies on the structures targeted by the flexible regions seen with PCA and FEL may help in the discovery of new inhibitors that capture the dynamics of EGFR1 to improve the treatment outcome of diseases like cancer.

### 3.10 Binding free energy analysis

**FIGURE 9:**
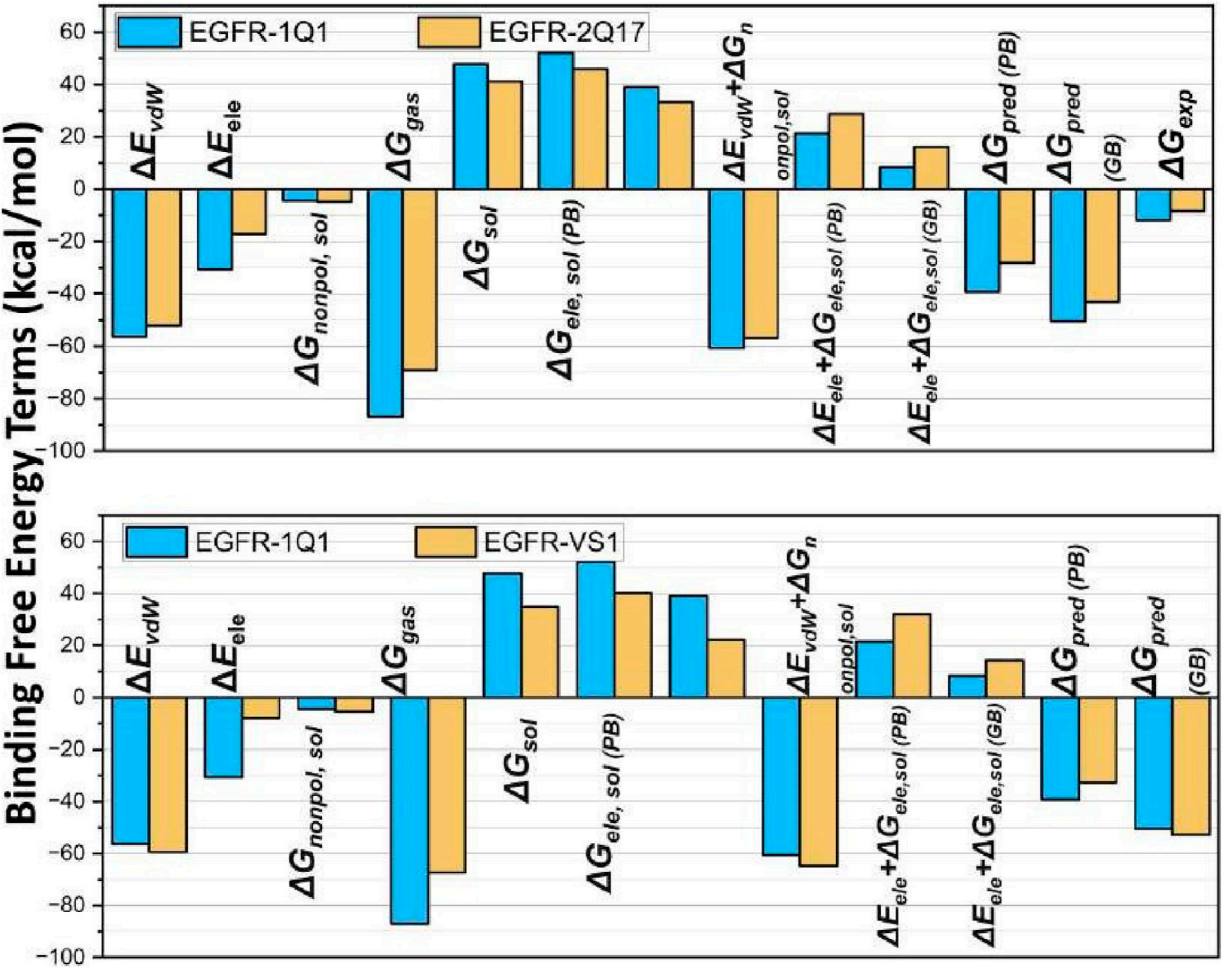
Comparison between binding free energy terms of EGFR1: **(A)** 1Q1 and 2Q17, and **(B)** 1Q1 and VS1. The plots show the differences in binding free energy components, providing insights into the molecular interactions and stability within each system.

Given the system stability ascertained through RMSD fluctuations depicted in Figures 6, 7, we extracted 10,000 snapshots randomly from 1 to 200 ns of the MD simulation for binding free energy calculations. The binding affinities of the chosen compounds towards EGFR1 were determined using both MM/PBSA and MM/GBSA methods. Although the binding free energies calculated with MM/PBSA and MM/GBSA were slightly higher than the absolute experimental values (ΔG_exp_), the ranking of the predicted binding affinities (ΔG_pred (GB)_) of inhibitors 1Q1, 2Q17, and VS1 (−50.54, −43.1132, and −52.8760 kcal/mol, respectively) aligned closely with their experimental IC50 values for EGFR1 (1Q1 IC50 = 2 nM; 2Q17 IC50 = 881 nM). This alignment, as detailed in Supplementary Table S4, highlights that 1Q1 and VS1 possess higher ΔGpred (GB/PB) values in the EGFR1-bonded systems, indicating a stronger binding affinity to the EGFR1 binding pocket. In contrast, 2Q17 displayed lower ΔG_pred (GB/PB)_ values, suggesting a reduced binding affinity towards EGFR1. These findings corroborate that the ΔG_pred_ (GB/PB) values derived from MM/GB/PB/SA methods are consistent with experimental observations, underscoring the effectiveness of our computational approach in predicting the binding efficiency of potential EGFR1 inhibitors. The MMGB/PBSA method’s ability to dissect the total binding free energy into its constituent components provides a nuanced understanding of the ligand-receptor binding dynamics. As detailed in Figure 9, the polar solvation energies (ΔEele, sol) exhibit positive values, indicating a counteractive effect against the favorable electrostatic energies (ΔEele) observed in the gas phase across all three complexes. This results in the combined electrostatic contributions (ΔGele+ΔGele, sol) being unfavorable for the formation of ligand-receptor complexes. Conversely, the van der Waals interactions and nonpolar solvation energy (ΔEvdW + ΔGnonpol,sol) contribute negative values, enhancing the binding affinity of the ligands to the receptor. Notably, the ΔEvdW values surpass the ΔEele term for all examined systems, underscoring the critical role of optimizing van der Waals and nonpolar interactions in augmenting the inhibitory effectiveness of EGFR1 inhibitors. The presence of several hydrophobic residues, such as L694, V702, A719, K721, Y777, L820, L830, and F832, aligns with the observation that hydrophobic interactions significantly influence binding efficiency. Despite the electrostatic contribution being less dominant compared to the van der Waals and nonpolar solvation contributions, it remains a pivotal factor in mediating interactions between EGFR1 and inhibitors like 1Q1. The electrostatic interactions particularly enhance the binding effects with ligands 1Q1 and VS1 more than with 2Q17. Analysis of the EGFR1 complexes further exemplifies the predominance of van der Waals interactions in modulating inhibitory potency, with the ΔEvdW term showcasing significant negative values for EGFR1-1Q1 (−56.256 kcal/mol), EGFR1-2Q17 (−52.09 kcal/mol), and VS1 (−59.39 kcal/mol) complexes. These findings emphasize that while electrostatic interactions contribute to the binding process, the van der Waals and hydrophobic interactions play a more substantial role in determining the inhibitory potential of EGFR1 inhibitors. This analysis not only sheds light on the binding mechanisms but also offers strategic directions for designing more potent EGFR1 inhibitors by focusing on enhancing van der Waals and hydrophobic interactions.

### 3.11 Binding modes of 1Q1, 2Q17 and VS1 in EGFR1-bounded systems

**FIGURE 10:**
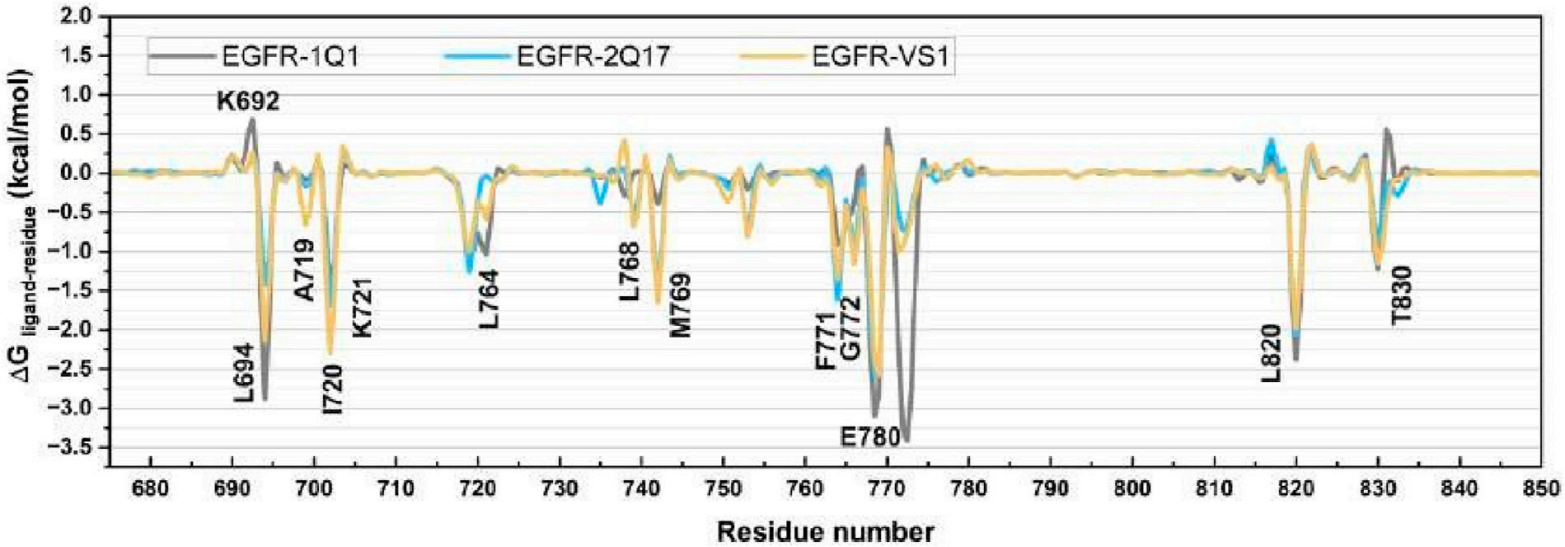
Comparison of per-residue energy decomposition (ΔGligand-residue) of EGFR1 bound to 1Q1, 2Q17, and VS1. The plot highlights the contribution of individual residues to the overall binding free energy, offering detailed insights into the key residues involved in stabilizing ligand interactions across the three complexes.

To pinpoint the critical residues involved in the ligand-receptor binding dynamics, we dissected the overall binding free energy, as calculated via the MM/GBSA method, into the contributions from individual residues. As illustrated in Figure 10, the binding of ligands 1Q1, 2Q17, and VS1 to EGFR1 is significantly enhanced by interactions with specific residues, notably L694, A719, I720, K721, L764, L768, M769, F771, G772, M780, L820, and T830. Interestingly, K692 across all three complexes did not contribute favorably to electrostatic interactions. In assessing the binding affinities of these ligands to EGFR1, our analysis focused on residues that showed substantial differences in their contributions to the binding free energies. Figure 10 highlights that residues L694, I720, G772, and E780 are pivotal for the superior binding affinity of 1Q1 compared to 2Q17. Additionally, in the VS1-EGFR1 complex, I720, L768, M769, and E780 emerge as key contributors to its notable inhibitory efficacy against EGFR1. While 2Q17 interacts with many of the same residues as 1Q1 and VS1, it fails to form exceptionally strong contacts with any specific residue, underscoring its lower binding efficiency. This detailed residue-by-residue analysis of binding free energy contributions provides valuable insights for the targeted design of quinazoline-based small-molecule inhibitors, highlighting the importance of specific amino acids in enhancing binding affinity to EGFR1. These findings offer a strategic framework for developing more potent inhibitors by focusing on key residue interactions within the EGFR1 binding site.

## 4 Conclusion

Quinazoline derivatives are recognized as potent inhibitors of EGFR1, a key target in cancer therapy. This study leverages an integrated computational approach, combining ligand-based 3D-QSAR, structure-based docking, molecular dynamics (MD) simulations, and binding free energy analyses, to elucidate the ligand-protein interactions crucial for identifying and optimizing novel EGFR1 inhibitors. By contrasting the interaction characteristics of 1Q1, the most effective EGFR1 inhibitor, with those of 2Q17, the least effective, we provide insights that could steer the development of potent FGFR1 inhibitors. Additionally, the top inhibitor from virtual screening underwent further evaluation. Employing a comprehensive computational toolkit, including 3D-QSAR modeling, flexible docking, DFT, and MD simulations complemented by per-residue energy decomposition, this research examines the differential interaction patterns of efficacious versus less effective EGFR1 inhibitors at the molecular level. High q2 and R2 values from our models suggest a reliable prediction of ligand activities against EGFR1. Our findings reveal that 1Q1 and VS1 form more advantageous contacts within the EGFR1 complex compared to 2Q17, highlighting their superior inhibitory action. MD simulations and energy analyses further demonstrate how the sulfonamide group in VS1, and a similar functional group in 1Q1, enable stable binding within EGFR1’s active site through hydrogen bonding, hydrophobic interactions, and van der Waals contacts with critical residues. The outcomes of this investigation provide a valuable framework for the rational design of new EGFR1 inhibitors, focusing on quinazoline analogs that exhibit optimal ADMET properties. This integrated computational strategy offers a robust foundation for future lead compound discovery and the enhancement of EGFR1 inhibitors.

## Data Availability

The original contributions presented in the study are included in the article/Supplementary Material, further inquiries can be directed to the corresponding authors.

## Abbreviations

3D-QSAR, Quantitative Structural Activity Relationship; CoMFA, Comparative Molecular Field Analysis; CoMSIA, Comparative Molecular Similarity Indices Analysis; MD, Molecular Dynamics; MMGBSA, Molecular Mechanics Generalized Born Surface Area; MMPBSA, Molecular Mechanics Poisson-Boltzmann Surface Area; BFE, Binding Free Energy; RMSD, Root Mean Square Deviation; RMSF, Root Mean Square Fluctuation; ADMET, Absorption, Distribution, Metabolism, Excretion, Toxicity.
