# Supplementary material for: Deciphering quinazoline derivatives’ interactions with EGFR: a computational quest for advanced cancer therapy through 3D-QSAR, virtual screening, and MD simulations
Source: Front Pharmacol. 2024 Oct 24;15:1399372. doi: 10.3389/fphar.2024.1399372 (PMC11540632; doi:10.3389/fphar.2024.1399372)
Supplement: Supplementary file 1 [file DataSheet1.pdf]

Deciphering Quinazoline Derivatives' Interactions with EGFR: A Computational Quest for Advanced Cancer Therapy through 3D-QSAR, Virtual Screening, and MD Simulations

Sirajudheen Anwar[1*], Jowaher Alanazi[1], Nafees Ahemad[2], Shafaq Raza[3], Tahir Ali Chohan*, Hammad Saleem[3*]

[1]Department of Pharmacology and Toxicology, College of Pharmacy, University of Ha'il, Saudi Arabia.

[2]Institute of Pharmaceutical Sciences (IPS), University of Veterinary and Animal Sciences (UVAS), Lahore, Pakistan.

Correspondence: si.anwar@uoh.edu.sa; tahir.chohan@uvas.edu.pk; Hammad Saleem hammad.saleem@uvas.edu.pk

Abstract

The epidermal growth factor receptor (EGFR) presents a crucial target for combatting cancer mortality. This study employs a suite of computational techniques, including 3D-QSAR, ligand-based virtual screening, molecular docking, fingerprinting analysis, ADME, and DFT-based analyses (MESP, HOMO, LUMO), supplemented by molecular dynamics simulations and MMGB/PBSA free energy calculations, to explore the binding dynamics of quinazoline derivatives with EGFR. With strong q2 and r2 values from CoMFA and CoMSIA models, our 3D-QSAR models reliably predict EGFR inhibitors' efficacy. Utilizing a potent model compound as a reference, an E-pharmacophore model was developed to sift through the eMolecules database, identifying 19 virtual screening hits based on ShapeTanimoto, ColourTanimoto, and TanimotoCombo scores. These hits, assessed via 3D-QSAR, showed pIC_{50} predictions consistent with experimental data. Our analyses elucidate key features essential for EGFR inhibition, reinforced by ADME studies that reveal favorable pharmacokinetic profiles for most compounds. Among the primary phytochemicals examined, potential EGFR inhibitors were identified. Detailed MD simulation analyses on three select ligands—1Q1, 2Q17, and VS1—demonstrated their stability and consistent interaction over 200 ns, with MM/GBSA values corroborating their docking scores and highlighting 1Q1 and VS1's superior EGFR1 affinity. These results position VS1 as an especially promising lead in EGFR1 inhibitor development, contributing valuable insights towards crafting novel, effective EGFR1 inhibitors.

Keywords: EGFR; Anti-cancer; virtual screening; simulations; 3D-QSAR; *In-silico*; fingerprinting.

1. Molecular Dynamics simulations

Hundred nanoseconds (ns) MD simulations were carried out for all complexes using SANDER module in AMBER12 software package with the ff99SB force field. At first, the ligands were subjected to energy minimization by applying AM1 method [15] with HF/6- 31G* basis set in Gaussian 09 program. The force field parameters for the ligands were assigned using general AMBER force field (GAFF) together with RESP charges. LEaP program from AMBER 20 was used to assign missing hydrogen atoms. Each system was then immersed into an octahedron box of TIP3P [16] water model with a minimum distance of 10 Å from the solute surface. In order to maintain the neutrality, Cl− and Na+ counter ions were added to the EGFR1-complex, respectively. Before running MD simulation, energy was minimized in 3 consecutive steps to avoid steric clashes. During the first step of minimization only the counter ions (Cl− or Na+) and water molecules were minimized while ligand and protein molecules were kept fixed. The next step of minimization was performed with unconstraint side chains and only the heavy atoms were kept frozen with a constraint force of 5.0 kcal·(mol·Å2) −1. In the final step unconstrained minimization of the whole system was executed. All three stages of minimizations were performed by applying 2500 steps of the steepest descent followed by 5000 steps of conjugate gradient method. In an NVT ensemble, the temperature of the system was gradually amplified from 0 to 300 K for 100ps and maintained at this point. Each system of protein-ligand complex was then subjected to equilibration process by applying the Langevin dynamics [17] with a collision frequency of 1 PS-1 and the force constant was set to 10 kcal/(mol·Å2). Finally, each of the protein-ligand complex systems was then underwent the molecular dynamics simulation using NPT ensemble at constant temperature of 300 K and 1atm pressure. The time integration step was set to 2 fs [40]. to constraint all bonds containing hydrogen bond atoms. The nonbonded electrostatic interactions were treated with particle-mesh-Ewald (PME) [18] method and a 10 Å cutoff. The conformations of MD simulation trajectories were collected at every 1ps and 2ps during equilibration runs and production MD, respectively. All MD simulations procedures were accomplished using the CARNAL, ANAL, and PTRAJ modules of AMBER 12.

1.1. Free energy calculation

The snapshots collected during MD production runs were further used for structural and energetic analysis of each protein-ligand complex using molecular mechanics based scoring method MM/PB(GB)SA [19, 20], implemented in AMBER 20. To compare the binding free energies of selected compounds in EGFR1 complexes, MM/GBSA calculations were executed to the total of 1000 snapshots extracted from the final 2 ns of the MD trajectories of each complex system. The binding free energy calculations for each molecular species, including ligand-receptor complex, free receptor, and free ligand were performed as the difference between the total free energy of ligand-protein complex (Gcom) and the sum of free energy of individual protein (Gpro) and individual ligand (Glig). It can be calculated as follow:

$$\Delta Gbind = \Delta H – T\Delta S = Gcom – (Gprot + Glig) \qquad (2)$$

The free energy of ligand-protein complex, protein, and ligand in above equation (Eq. 2) can be calculated by the following equation:

$$\Delta G = \Delta GMM + \Delta Gsol – T\Delta S \qquad (3)$$

In Eq. 3, the total molecular mechanic energy in gas phase, solvation free energy, and entropy terms are denoted by GEMM, ΔGsol, and TΔS, respectively, at given temperature T.

In Eq. (4), ΔEMM is the sum of internal energies (ΔEint), van der Waals energies (ΔEvdW), and non-bonded electrostatic energies (ΔEele). While in Eq. (5), the solvation-free energy (ΔGsol) can be calculated as the sum of polar and nonpolar parts.

ΔEMM = ΔEint + ΔEvdW + ΔEele (4)

and,

ΔGsol = ΔGele, sol PB(GB) + ΔGnonpol; sol (5)

where the electrostatic contribution to solvation free energies (ΔGele,sol energies) can be computed by solving the Poisson_Boltzmann (PB) equations [21] or generalized Born (GB) model [22] for MMGBSA and MMPBSA methods. Linear combination of pairwise overlaps (LCPO) [23, 24] approach was applied to calculate the solvent accessible surface area (SASA, Å2) [23, 25]. Furthermore, molsurf module implicated in amber was used to determine the nonpolar solvation contributions ΔGnonpol,sol, as shown in Eq. (6):

ΔGnonpol;sol = γSASA + b (6)

where surface tension (0.0072 kcal·(mol·Å2)−1 is denoted by γ and the b is a constant (0).

The experimentally reported inhibitory activities in term of IC50 values were converted to the experimental binding free energies [27] using the following equation:

ΔGexp≈−RT·lnIC50 (8)

Where R=1.986×10−3 kcal/(K·mol), T=300 K, and Ki is in molar/mol.

1.2. Per-residue free energy decomposition analysis

All decomposition analysis were performed using MMGBSA module of AMBER20 [19, 20]. Pairwise the nature of the GB equation provides an opportunity to decompose free-energies into insightful interaction and desolation components. Hence, the ligand-protein interaction energies were further decomposed into van der Waals (ΔGvdw), electrostatic (ΔGele), polar (ΔGele, sol), and non-polar (ΔGnonpol, sol) contributions. It is described in equation 8 as mentioned equation.

ΔGinhibitor_residue = ΔGvdW + ΔGele + ΔGele, sol + ΔGnonpol, sol (9)

Sander module in AMBER12 software package was used to estimate the Van Der Waals (vdW) and electrostatic energy contributions in complex formation [20, 22]. All decomposition analysis were executed using the same snapshots which were used in previous calculations.

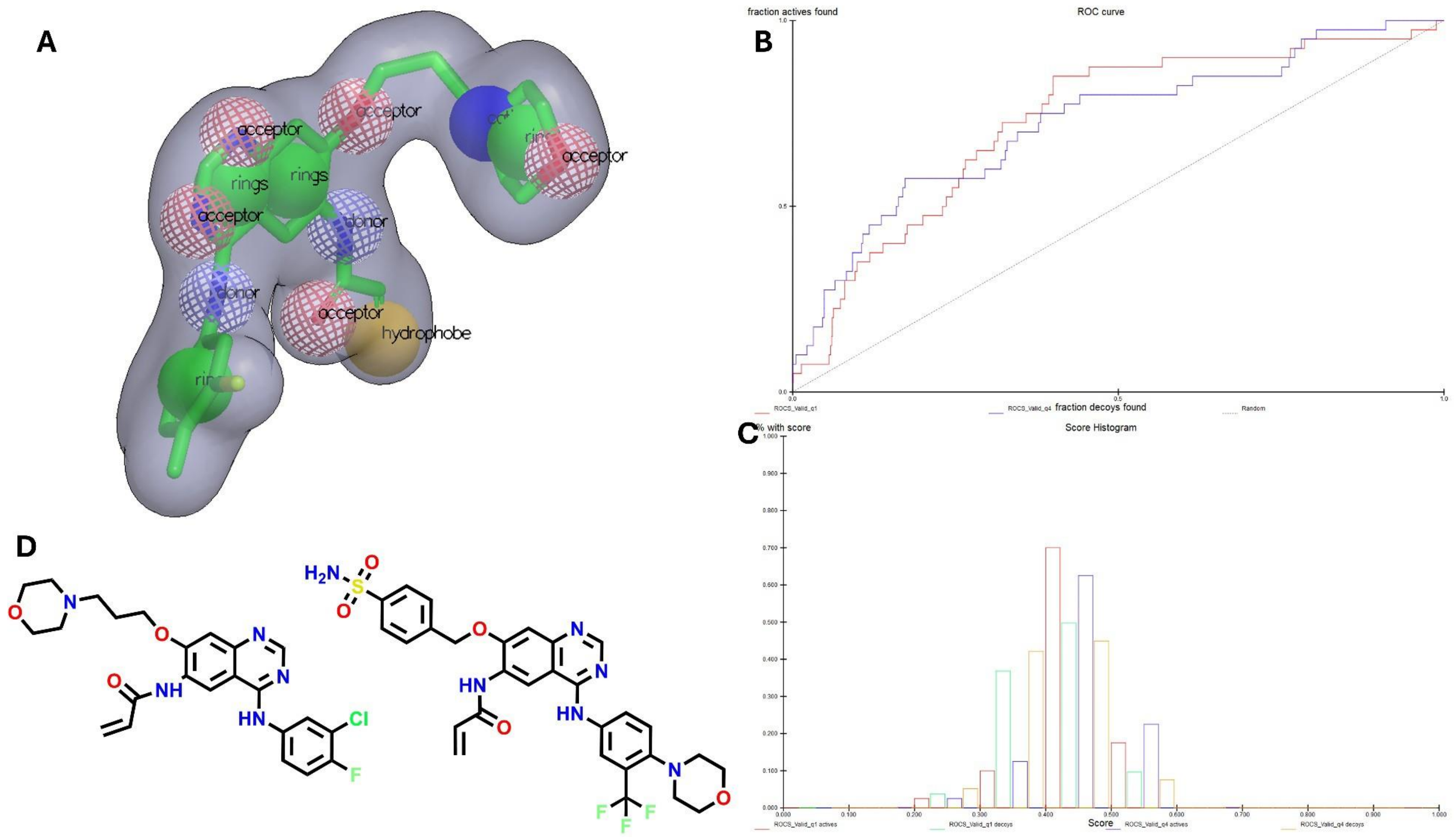

Figure S1. Ligand-based pharmacophoric query model and its validation; (A) query model of the co-crystal ligand (Ligand-based pharmacophoric features) generated from the reported pdf ID; (B) Tanimoto-Combo AUC-ROC curve for the validation of query model; (C) Shape-Tanimoto histogram for the validation of query model. (D) Chemical structure of the co-crystal ligand.

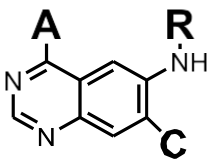

1Q

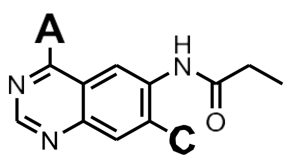

2Q

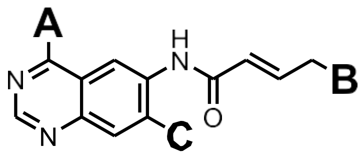

3Q

Table SP1. Structures, experimental and predicted inhibitory activities of the 3D-QSAR modeling data sets and docking scores.

Code	COMP#	R	A	B	C	IC_{50} (nM)	pIC_{50} (nM)	COMFA		COMSIA		DOCKING SCORES
								Pred	Res	Pred	Res	
	Training Set											
1Q1	4	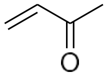	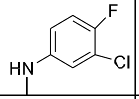	-	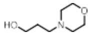	2	8.699	8.683	0.016	8.878	0.179	-8.32
1Q2	10	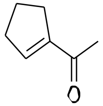	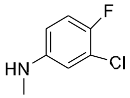	-	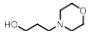	7	8.155	8.107	0.048	8.080	0.075	-8.148
2Q3	12	-	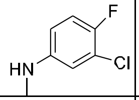	-	OCH3	2	8.699	8.693	0.006	8.027	0.672	-8. 225
2Q4	13	-	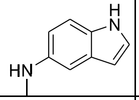	-	OCH3	6	8.222	8.190	0.032	8.253	0.031	-7.594
2Q5	14	-	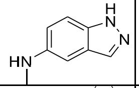	-	OCH3	79	7.102	7.138	-0.036	7.464	-0.362	-8.285
2Q6	15	-	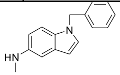	-	OCH3	21	7.678	7.707	-0.029	7.531	0.147	-7.547

2Q7	16	-	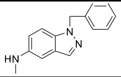	-	OCH3	17	7.770	7.722	-0.153	7.657	-0.175	-6.863
2Q8	17	-	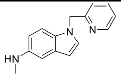	-	OCH3	61	7.215	7.028	0.045	6.882	-0.248	-6.819
2Q9	18	-	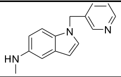	-	OCH3	714	6.146	6.206	0.052	6.831	-0.260	-7.17
2Q10	20	-	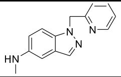	-	OCH3	94	7.027	7.073	-0.087	7.100	0.329	-6.55
2Q11	21	-	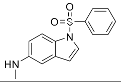	-	OCH3	127	6.896	6.852	-0.130	6.518	0.146	-5.110
2Q12	23	-	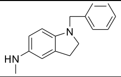	-	OCH3	175	6.757	6.843	0.002	6.600	0.303	-
2Q13	25	-	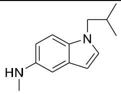	-	OCH3	421	6.376	6.397	0.023	6.324	-0.002	-6.023
2Q14	26	-	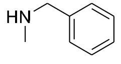	-	OCH3	136	6.866	6.899	0.073	7.105	0.207	-6.402
2Q15	27	-	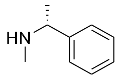	-	OCH3	86	7.066	7.025	0.058	6.956	0.265	-7.575
2Q16	28	-	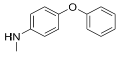	-	OCH3	65	7.187	7.168	0.04	7.514	-0.013	-6.116
2Q17	29	-	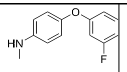	-	OCH3	881	6.055	6.180	0.051	6.213	0.003	-4.46
2Q18	30	-	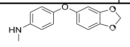	-	OCH3	509	6.293	6.199	-0.058	6.151	-0.111	-5.198
2Q19	34	-	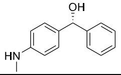	-	OCH3	343	6.465	6.535	0.099	6.449	0.016	-4.876
2Q20	35	-	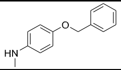	-	OCH3	36	7.444	7.540	-0.132	7.583	-0.180	-5.188
2Q21	37		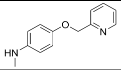	-	OCH3	116	6.936	7.029	0.109	7.286	0.081	-5.984

2Q22	39	-	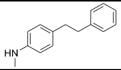	-	OCH3	747	6.127	6.085	-0.016	6.002	-0.084	-6.001
2Q23	40	-	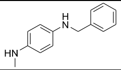	-	OCH3	383	6.417	6.282	0.140	6.344	0.147	-5.612
2Q24	41	-	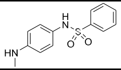	-	OCH3	65	7.187	7.104	-0.102	7.08	-0.247	-4.001
2Q25	42	-	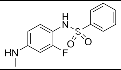	-	OCH3	593	6.227	6.283	0.035	6.251	-0.019	-4.996
2Q26	43	-	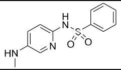	-	OCH3	522	6.282	6.307	-0.001	6.274	-0.038	-5.013
3Q27	44	-	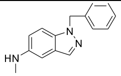	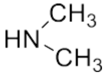	OC2H5	6	8.222	8.033	0.104	7.759	-0.281	-6.182
3Q28	46	-	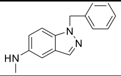	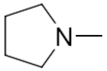	OC2H5	7	8.155	7.960	-0.049	7.918	0.150	-6.538
3Q29	47		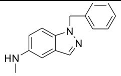	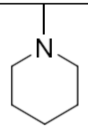	OC2H5	8	8.097	7.926	0.07	7.862	0.079	-6.708
3Q30	49		- 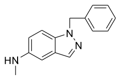	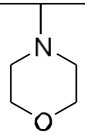	OC2H5	26	7.585	7.904	0.006	7.861	-0.274	-4.972
3Q31	50	-	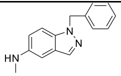	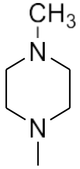 -	OC2H5	23	7.638	7.873	0.038	7.780	-0.024	-3.500
3Q32	52		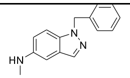 -	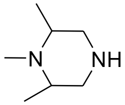	OC2H5	72	7.143	7.030	-0.073	7.028	-0.295	-6.001
3Q33	54	-	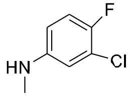	- 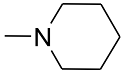	OCH3	6	8.222	8.232	-0.09	8.370	-0.340	-5.086

3Q34	55	-	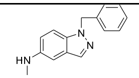	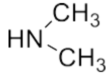	$HO-\underset{H_2}{C}-\overset{H_2}{C}-OCH_3$	7	8.155	8.165	0.011	7.881	-0.187	-7.154
3Q35	56	-	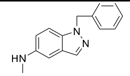	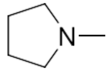	$HO-\underset{H_2}{C}-\overset{H_2}{C}-OCH_3$	10	8	8.083	0.033	8.029	0.223	-7.100
3Q36	57	-	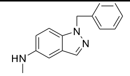	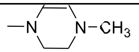	$HO-\underset{H_2}{C}-\overset{H_2}{C}-OCH_3$	16	7.796	7.850	0.246	7.747	0.526	-6.143
3Q37	59	-	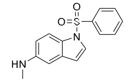	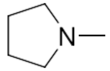	$HO-CH_2-CH_3$	36	7.444	7.472	0.076	7.197	0.283	-7.710
3Q38	60	-	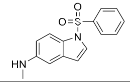	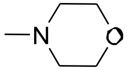	$HO-CH_2-CH_3$	122	6.914	6.823	-0.148	7.047	0.025	-6.889
3Q39	61	-	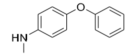	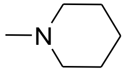	$HO-CH_3$	132	6.879	6.842	-0.154	6.957	-0.140	-7.008
3Q40	62	-	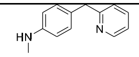	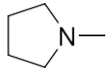	$HO-CH_3$	148	6.830	6.853	-0.074	6.927	-0.169	-6.760
3Q41	63	-	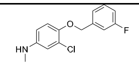	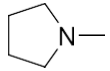	$HO-CH_2-CH_3$	60	7.222	7.307	-0.017	7.312	-0.067	-5.590
3Q42	64	-	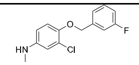	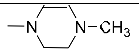	$HO-CH_2-CH_3$	23	7.638	7.602	0.013	7.523	0.024	-7.139
3Q43	65	-	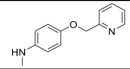	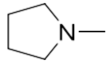	$HO-CH_2-CH_3$	98	7.009	7.092	-0.168	7.398	-0.286	-5.716
3Q44	68	-	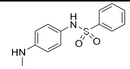	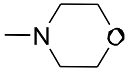	$HO-CH_2-CH_3$	69	7.161	7.067	-0.177	7.235	-0.130	-5.440
3Q45	69	-	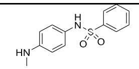	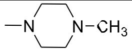	$HO-CH_2-CH_3$	82	7.086	7.099	0.448	7.285	-0.05	-1.871
Test set												
1Q46	8	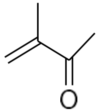	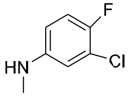	-	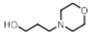	21	6.127	6.268	-0.141	6.449	-0.322	-7.624.
1Q47	9	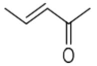	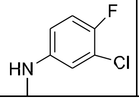	-	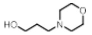	22	6.055	6.404	-0.349	7.702	-1.647	-7.259

1Q48	11	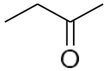	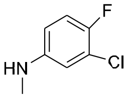	-	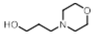	9	8.046	7.567	0.479	7.394	0.652	-8.214
2Q49	22	-	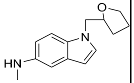	-	OCH3	86	7.066	7.669	-0.603	7.011	0.055	-5.881
2Q50	24	-	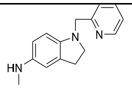	-	OCH3	519	7.187	7.568	-0.381	7.016	0.141	-5.770
2Q51	31	-	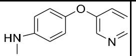	-	OCH3	531	6.275	7.553	-1.278	6.775	-0.50	-5.771
2Q52	33	-	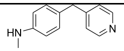	-	OCH3	388	6.411	7.551	-1.14	6.757	-0.346	-6.151
2Q53	36	-	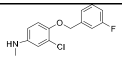	-	OCH3	67	7.174	7.570	-0.396	6.900	0.144	
2Q54	32	-	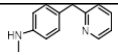	-	OCH3	205	8.222	7.530	0.692	7.579	0.643	-6.182
3Q55	45		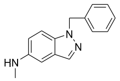	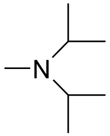	$HO-CH_2-CH_3$	15	7.824	7.537	0.287	7.917	-0.093	-5.667
3Q56	48		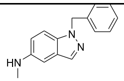	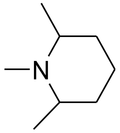	$HO-CH_2-CH_3$	79	7.102	7.556	-0.454	7.887	-0.785	-4.323
3Q57	51		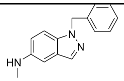	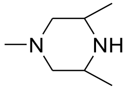	$HO-CH_2-CH_3$	17	7.770	7.550	0.220	7.883	-0.113	-5.678

3Q58	53	-	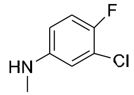 -	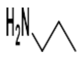	$HO-CH_3$	2	8.699	7.568	1.131	7.766	0.933	-4.543
3Q59	58	-	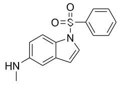	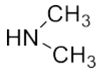	$HO-CH_2-CH_3$	38	7.420	7.570	-0.150	7.552	-0.132	-5.543
3Q60	66	-	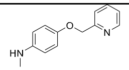	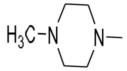	$HO-CH_2-CH_3$	164	6.785	7.569	-0.784	7.728	-0.943	-5.004
3Q61	67	-	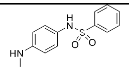	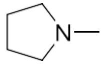	$HO-CH_2-CH_3$	21	7.678	7.520	0.158	7.735	-0.057	-4.997
VS1		-	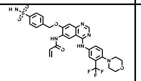				-	9.534	-	9.342		-12.961
VS2							-	9.423	-	8.954		-12.954
VS3			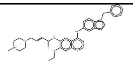				-	8.952	-	8.874		-12.313
VS4			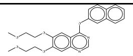				-	8.91	-	8.543		-12.255
VS5			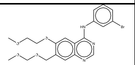				-	8.132	-	8.012		-9.746
VS6			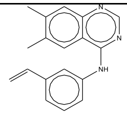				-	8.101	-	7.987		-9.743

VS7			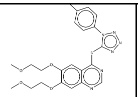				-	8.321	-	7.935		-9.702
VS8			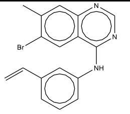				-	8.453	-	8.021		-9.449
VS9			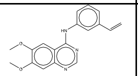				-	8.221	-	8.005		-9.426
VS10			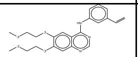				-	8.322	-	7.554		-9.401
VS11			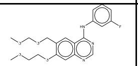				-	8.175	-	7.882		-9.391
VS12			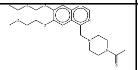				-	8.534	-	8.238		-9.374
VS13			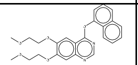				-	8.546	-	7.554		-9.28
VS14			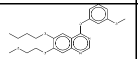				-	8.657	-	7.432		-9.271
VS15			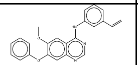				-	8.95	-	8.213		-9.243
VS16			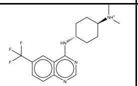				-	7.578	-	7.021		-9.127
VS17			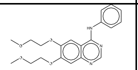				-	8.295	-	8.012		-9.114

VS18			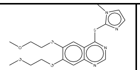				-	8.865	-	7.832		-9.09
VS19			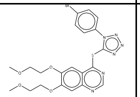				-	8.884	-	7.833		-9.00

IC_{50}: Half-maximal inhibitory concentration, Pred: Predicted IC_{50} values, pIC_{50}: Negative log of IC_{50} Res: Difference between actual and predicted IC_{50} values, Docking score =Glide docking score. Pred: Predicted IC_{50} values, Docking score = Glide docking score.

Table S2: The area under ROC curves (AUC), enrichment factor (is 0.5 %, 1 % and 2 %) of the 3D virtual screening protocols for selecting query model.

Sr no	Statistical Matrices	Query conformer 2	Query conformer 4	Query conformer 5
**ROCS_TanimotoCombo**				
1	**AUC**	0.732	0.718	0.728
2	**0.5 % Enrichment**	9.929	19.683	17.417
3	**1.0 % Enrichment**	5.380	12.427	9.831
4	**2.0 % Enrichment**	3.714	6.389	5.484

Table SP2. Pharmacokinetic properties of model compounds.

Sr. No.	Compounds	Pharmacokinetics									Synthetic accessibility
		Absorption	Metabolism						Excretion	Toxicity	
		Intestinal absorption (Human)	CYP						Total Clearance	AMES Toxicity	
		Numeric (% absorbed)	2D6 Sub	3A4 sub	1A2 inh	2C19 Inh	2C9 inh	2D6 inh	3A4 inh	Numeric (log ml/min/kg)	Categorical (yes/no)	Numeric
1	1Q1	90.396	No	Yes	No	No	No	No	Yes	0.859	No	3.48
2	1Q2	89.739	No	Yes	No	No	No	No	Yes	1.022	No	3.91
3	2Q3	92.633	No	No	Yes	Yes	Yes	No	Yes	0.346	No	2.68
4	2Q4	89.69	No	No	Yes	Yes	Yes	No	Yes	0.419	No	2.70
5	2Q5	83.219	No	No	Yes	Yes	Yes	No	Yes	0.214	No	2.70

6	2Q6	95.982	No	Yes	Yes	Yes	Yes	No	Yes	0.524	No	3.15
7	2Q7	97.13	No	Yes	Yes	Yes	Yes	No	Yes	0.249	No	3.20
8	2Q8	97.235	No	Yes	No	Yes	Yes	No	Yes	0.488	No	3.22
9	2Q9	97.187	No	Yes	Yes	Yes	Yes	No	Yes	0.568	No	3.24
10	2Q10	92.636	No	Yes	Yes	No	Yes	No	Yes	0.269	No	3.28
11	2Q11	93.801	No	Yes	No	Yes	Yes	No	Yes	0.592	No	3.43
12	2Q12	95.588	No	Yes	Yes	Yes	Yea	No	Yes	0.273	No	3.32
13	2Q13	94.38	No	Yes	Yes	Yes	Yes	No	Yes	0.541	No	3.14
14	2Q14	93.13	No	No	Yes	Yes	Yes	No	Yes	0.477	No	2.40
15	2Q15	93.133	No	No	Yes	Yes	Yes	No	Yes	0.459	No	2.95
16	2Q16	92.745	No	Yes	Yes	Yes	Yes	No	Yes	0.249	No	3.04

17	2Q17	92.87	No	Yes	No	Yes	Yes	No	Yes	0.151	No	3.14
18	2Q18	100	No	Yes	No	Yes	Yes	No	Yes	0.093	No	3.48
19	2Q19	88.016	No	Yes	Yes	Yes	Yes	No	Yes	0.219	No	3.51
20	2Q20	92.941	No	Yes	No	Yes	Yes	No	Yes	0.254	No	3.12
21	2Q21	89.925	No	Yes	No	Yes	Yes	No	Yes	0.627	No	3.19
22	2Q22	93.468	No	Yes	Yes	Yes	Yes	No	Yes	0.311	Yes	3.09
23	2Q23	91.162	No	Yes	Yes	Yes	Yes	No	Yes	0.601	No	3.01
24	2Q24	83.677	No	Yes	No	Yes	Yes	No	Yes	0.461	No	3.33
25	2Q25	85.683	No	Yes	No	Yes	Yes	No	Yes	0.057	No	3.40
26	2Q26	75.741	No	Yes	No	Njo	Yes	No	Yes	0.191	No	3.59
27	3Q27	100	No	Yes	No	Yes	No	No	Yes	0.706	No	3.88

28	3Q28	100	No	Yes	No	Yes	No	No	Yes	0.88	No	4.02
29	3Q29	100	No	Yes	No	Yes	No	No	Yes	0.832	No	4.13
30	3Q30	100	No	Yes	No	Yes	No	No	Yes	0.874	No	4.14
31	3Q31	100	No	Yes	No	Yes	Yes	No	Yes	0.641	No	4.29
32	3Q32	100	No	Yes	No	Yes	Yes	No	Yes	0.605	No	5.22
33	3Q33	92.643	No	Yes	No	No	No	No	Yes	0.674	No	3.41
34	3Q34	100	No	Yes	No	Yes	No	No	Yes	0.73	No	4.10
35	3Q35	100	No	Yes	No	Yes	No	No	Yes	0.903	No	4.23
36	3Q36	96.878	No	Yes	No	Yes	No	No	Yes	0.664	No	4.50
37	3Q37	97.412	No	Yes	No	Yes	No	No	Yes	0.437	No	4.21
38	3Q38	96.359	No	Yes	No	No	No	No	Yes	0.431	No	4.35

39	3Q39	92.578	No	Yes	No	Yes	Yes	No	Yes	0.558	No	3.84
40	3Q40	100	No	Yes	No	No	Yes	No	Yes	0.917	No	3.72
41	3Q41	92.096	No	Yes	No	Yes	Yes	No	Yes	0.599	No	4.08
42	3Q42	99.408	No	Yes	No	No	No	No	Yes	0.369	No	4.36
43	3Q43	100	No	Yes	No	No	Yes	No	Yes	0.954	No	3.96
44	3Q44	95.065	No	Yes	No	Yes	No	No	Yes	0.47	No	4.26
45	3Q45	86.584	No	Yes	No	No	No	No	Yes	0.246	No	4.41
46	1Q46	91.141	No	Yes	No	No	Yes	No	Yes	0.808	No	3.61
47	1Q47	91.179	No	Yes	No	No	Yes	No	Yes	0.76	No	3.65
48	1Q48	91.054	No	Yes	No	No	Yes	No	Yes	0.892	No	3.51
49	2Q49	94.604	No	Yes	Yes	Yes	Yes	No	Yes	0.526	No	3.79

50	2Q50	95.047	No	Yes	Yes	Yes	Yes	No	Yes	0.216	No	3.38
51	2Q51	91.825	No	No	Yes	No	Yes	No	Yes	0.25	No	3.19
52	2Q52	95.092	No	Yes	Yes	Yes	Yes	No	Yes	0.311	No	2.98
53	2Q53	92.123	No	Yes	No	Yes	Yes	No	Yes	0.162	No	3.31
54	2Q54	95.433	No	Yes	Yes	Yes	Yes	No	Yes	0.407	No	N
55	3Q55	100	No	Yes	No	Yes	No	No	Yes	0.821	No	4.38
56	3Q56	100	No	Yes	No	Yes	No	No	Yes	0.806	No	5.10
57	3Q57	100	No	Yes	No	Yes	Yes	No	Yes	0.569	No	5.10
58	3Q58	92.116	No	Yes	No	No	Yes	No	Yes	0.697	No	3.31
59	3Q59	91.525	No	Yes	Yes	Yes	No	No	Yes	0.262	No	4.10
60	3Q60	92.814	No	Yes	No	No	Yes	No	Yes	0.724	No	4.25

61	3Q61	96.119	No	Yes	No	Yes	No	No	Yes	0.476	No	4.12
62	VS1	99.812	NO	YES	NO	YES	YES	NO	YES	-0.185	NO	
63	VS2	96.568	Yes	Yes	No	Yes	Yes	Yes	Yes	1.28	Yes	
64	VS3	98.493	No	Yes	No	Yes	No	No	Yes	0.871	No	
65	VS4	97.519	No	Yes	Yes	Yes	Yes	No	Yes	0.856	No	
66	VS5	93.574	No	No	No	Yes	Yes	No	Yes	0.519	No	
67	VS6	93.14	No	No	Yes	Yes	No	No	Yes	0.419	No	
68	VS7	100	No	Yes	No	No	No	No	Yes	0.652	No	
69	VS8	91.614	No	No	Yes	Yes	No	No	Yes	-0.027	No	
70	VS9	94.371	No	No	Yes	No	Yes	No	Yes	0.411	No	
71	VS10	96.471	No	Yes	Yes	Yes	Yes	No	Yes	0.486	No	

72	VS11	94.688	No	No	No	Yes	Yes	No	Yes	0.448	No	
73	VS12	96.158	No	Yes	No	No	No	No	No	0.995	No	
74	VS13	98.215	No	Yes	Yes	Yes	Yes	No	Yes	0.913	No	
75	VS14	97.537	No	Yes	Yes	Yes	Yes	No	No	0.93	No	
76	VS15	95.694	No	Yes	Yes	Yes	Yes	No	Yes	0.316	Yes	
77	VS16	93.771	No	No	Yes	No	No	No	No	1.011	No	
78	VS17	94.66	No	No	Yes	Yes	Yes	No	Yes	0.537	No	
79	VS18	97.539	No	No	Yes	No	Yes	No	No	0.733	No	
80	VS19	100	No	Yes	Yes	No	Yes	No	Yes	0.456	No	

Table S4. Binding free energy calculations of inhibitory molecules for the target protein EGFR.

**Complex**	**EGFR-1Q1**	**AEGFR-2Q17**	**EGFR-VS1**
$\Delta E_{vdW}{}^{a}$	-56.2562	-52.0968	-59.3939
$\Delta E_{\text{ele}}{}^{a}$	-30.676	-17.108	-7.9898
$\Delta G_{\text{nonpol, sol}}{}^{a}$	-4.4327	-4.8519	-5.3344
ΔG_{gas}	-86.9322	-69.2048	-67.3837
ΔG_{sol}	47.6495	41.0951	34.7830
$\Delta G_{\text{ele, sol (PB)}}{}^{a}$	52.0821	45.947	40.1174
$\Delta G_{\text{ele, sol (GB)}}{}^{a}$	39.0250	33.1604	22.1590
$\Delta E_{vdW}+\Delta G_{\text{nonpol,sol}}{}^{a}$	-60.6889	-56.9487	-64.7283
$\Delta E_{\text{ele}}+\Delta G_{\text{ele,sol (PB)}}{}^{a}$	21.4061	28.839	32.1276
$\Delta E_{\text{ele}}+\Delta G_{\text{ele,sol (GB)}}{}^{a}$	8.36	16.06	14.17
$\Delta G_{\text{pred (PB)}}{}^{b}$	-39.2827	-28.1097	-32.6007
$\Delta G_{\text{pred (GB)}}{}^{b}$	−50.54	-43.1132	-52.8760
$IC_{50(\text{nM})}{}^{c}$	2	9870	NA
$\boldsymbol{\Delta G_{\text{exp}}{}^{d}}$	-11.86	-8.26	NA

[a]All energies are in kcal/mol, ΔH: the enthalpy changes, $\Delta H = G_{\text{ele}} + \Delta G_{vdW} + \Delta G_{\text{nonpol,sol}} + \Delta G\text{ele,sol}$. [b] ΔG_{pred}: the calculated binding free energy by the MMPB(GB)SA method. [c] Ki values of 3A, 4B, and 5B were taken from ref. 18. [d] ΔGexp: the experimental binding free energy was calculated according to the IC_{50} by $\Delta G \approx RT\ln(K_d)$
